# Transmission of *Vibrio cholerae* Is Antagonized by Lytic Phage and Entry into the Aquatic Environment

**DOI:** 10.1371/journal.ppat.1000187

**Published:** 2008-10-24

**Authors:** Eric J. Nelson, Ashrafuzzaman Chowdhury, James Flynn, Stefan Schild, Lori Bourassa, Yue Shao, Regina C. LaRocque, Stephen B. Calderwood, Firdausi Qadri, Andrew Camilli

**Affiliations:** 1 Howard Hughes Medical Institute and the Department of Molecular Biology and Microbiology, Tufts University School of Medicine, Boston, Massachusetts, United States of America; 2 Microbiology Department, Jahangirnagar University, Savar, Dhaka, Bangladesh; 3 Tufts Expression Array Core (TEAC) Facility, Tufts University School of Medicine, Boston, Massachusetts, United States of America; 4 Institut fuer Molekulare Biowissenschaften, Karl-Franzens-Universitaet Graz, Graz, Austria; 5 Division of Infectious Diseases, Massachusetts General Hospital, Boston, Massachusetts, United States of America, and Harvard Medical School, Boston, Massachusetts, United States of America; 6 International Centre for Diarrhoeal Disease Research, Dhaka, Bangladesh; Massachusetts General Hospital, United States of America

## Abstract

Cholera outbreaks are proposed to propagate in explosive cycles powered by hyperinfectious *Vibrio cholerae* and quenched by lytic vibriophage. However, studies to elucidate how these factors affect transmission are lacking because the field experiments are almost intractable. One reason for this is that *V. cholerae* loses the ability to culture upon transfer to pond water. This phenotype is called the active but non-culturable state (ABNC; an alternative term is viable but non-culturable) because these cells maintain the capacity for metabolic activity. ABNC bacteria may serve as the environmental reservoir for outbreaks but rigorous animal studies to test this hypothesis have not been conducted. In this project, we wanted to determine the relevance of ABNC cells to transmission as well as the impact lytic phage have on *V. cholerae* as the bacteria enter the ABNC state. Rice-water stool that naturally harbored lytic phage or *in vitro* derived *V. cholerae* were incubated in a pond microcosm, and the culturability, infectious dose, and transcriptome were assayed over 24 h. The data show that the major contributors to infection are culturable *V. cholerae* and not ABNC cells. Phage did not affect colonization immediately after shedding from the patients because the phage titer was too low. However, *V. cholerae* failed to colonize the small intestine after 24 h of incubation in pond water—the point when the phage and ABNC cell titers were highest. The transcriptional analysis traced the transformation into the non-infectious ABNC state and supports models for the adaptation to nutrient poor aquatic environments. Phage had an undetectable impact on this adaptation. Taken together, the rise of ABNC cells and lytic phage blocked transmission. Thus, there is a fitness advantage if *V. cholerae* can make a rapid transfer to the next host before these negative selective pressures compound in the aquatic environment.

## Author Summary

The biological factors that control the transmission of water-borne pathogens like *Vibrio cholerae* during outbreaks are ill defined. In this study, a molecular analysis of the active but non-culturable (ABNC) state of *V. cholerae* provides insights into the physiology of environmental adaptation. The ABNC state, lytic phage, and hyperinfectivity were concurrently followed as *V. cholerae* passaged from cholera patients to an aquatic reservoir. The relevance to transmission of each factor was weighed against the others. As the bacteria transitioned from the patient to pond water, there was a rapid decay into the ABNC state and a rise of lytic phage that compounded to block transmission in a mouse model. These two factors give reason for *V. cholerae* to make a quick transit through the environment and onto the next human host. Thus, in over-crowded locations with failed water infrastructure, the opportunity for fast transmission coupled with the increased infectivity and culturability of recently shed *V. cholerae* creates a charged setting for explosive cholera outbreaks.

## Introduction

Diarrheal disease is the second most common cause of death among children under 5 years of age globally – it is the leading cause of morbidity [1],[2]. The Gram-negative bacterium *Vibrio cholerae* is a facultative pathogen having both human and environmental stages, and is the etiologic agent of the secretory diarrheal disease cholera [3]. Today, the burden of cholera is estimated to reach several million cases a year in both Asia and Africa, with fewer cases in Latin America [4]. Aquatic reservoirs harbor *V. cholerae* during extended periods between outbreaks [5], but there is little known about how fast *V. cholerae* moves from one patient to the next during an outbreak. Transmission between patients may be quite rapid. For example, two devastating outbreaks strike Dhaka, Bangladesh annually. The high burden of disease [6], collapsed water infrastructure, poverty, and crowding make Dhaka an ideal setting for the fast transmission of a facultative pathogen such as *V. cholerae*. At the host population level, first degree relatives in households are more likely to be infected with *V. cholerae* [7]. At the pathogen level, the di-annual cholera outbreaks may be clonal [8],[9],[10], and there are rapid shifts in drug resistance patterns [11],[12]. Despite these epidemiological observations that support a model for rapid transmission during an outbreak, little is known about the selective forces that drive facultative pathogens – like *V. cholerae* – out of the environment and into the next host.

Using the infant-mouse model of cholera, we recently demonstrated that genes induced late in the infection provide a fitness advantage for the transition to aquatic environments [13]. In this study, *V. cholerae* from cholera patients or *in vitro* culture were transferred to an aquatic environment. We tested three factors as potential selective forces for driving *V. cholerae* out of the aquatic environment and into the next host. These factors are shared among several facultative pathogens and are as follows: the viable but non-culturable state, hyperinfectivity, and lytic phage. *Escherichia coli*, *Shigella sonnei*, *Listeria monocytogenes*, *Campylobacter jejuni*, and *V. cholerae* are examples of facultative pathogens that lose the ability to culture on standard media upon transfer to aquatic environments [14],[15]. This phenotype was traditionally called the viable but non-culturable state (VBNC) because the cells maintain the capacity for metabolic activities such as protein synthesis, respiration, and have intact membranes despite their inability to culture [16]. However, we prefer to use the active but non-culturable (ABNC) term for reasons explained by Kell *et al* [17]. The critical debate over terminology is if it is possible for bacteria with a known *in vitro* growth condition to be viable and (but) nonculturable. Since the answer to this question seems unresolved, the ABNC term is a more conservative definition. In the case of *V. cholerae*, animals become infected when inoculated with high doses of ABNC bacteria (>10^6^ or >1000-fold above the typical ID_50_ in animal models) suggesting that ABNC bacteria can be rescued for vegetative growth *in vivo* [14]. The experimental designs in these studies were unfortunately not overly relevant to conditions in the field; the ABNC state was induced by prolonged incubation at 4°C. ABNC *V. cholerae* have been observed in rural and urban water samples in Bangladesh between and during outbreaks [5]. ABNC *V. cholerae* are found as single cells or associated in aggregates with phytoplankton and zooplankton [5],[18],[19],[20]. For these reasons, ABNC *V. cholerae* are proposed to be the environmental reservoir that maintains *V. cholerae* between outbreaks and seeds new outbreaks. However, the role this reservoir plays during an outbreak is unclear because cholera outbreaks accelerate faster than the stochastic contribution of *V. cholerae* from an environmental reservoir [21].

The second factor we measured was hyperinfectivity. This phenotype was discovered when *V. cholerae* from patients were found to be more infectious in the infant-mouse model than *in vitro* grown *V. cholerae* [22],[23]. This phenotype has also been documented in *Citrobacter rodentium* [24], and can be modeled with mouse passaged bacteria [25]. The role hyperinfectivity plays in transmission is largely unknown, but *V. cholerae* from patients remain hyperinfectious for at least 5 h in pond water [23]. Models suggest that outbreaks start when an index case consumes *V. cholerae* from an environmental reservoir, but the acceleration of the outbreak is driven by hyperinfectious *V. cholerae*. Unlike the stochastic contribution of environmental *V. cholerae*, mathematical models that incorporate hyperinfectivity produce the steep rise in case numbers that are consistent with the actual rise in cases observed in Dhaka, Bangladesh during an outbreak [21].

The third factor we examined was lytic phage; we note here that this report concerns only lytic vibriophage and not cholera toxin phage or other lysogenic phage. Lytic vibriophage in the environment have been studied from almost the time that *V. cholerae* was first discovered [26], but recent phage epidemiology papers provide new insights into the role phage play in outbreaks. The percentage of patients passing lytic phage rises as a cholera outbreak progresses; at the same time, phage titers in the environment increase [27],[28]. Towards the end of an outbreak, the vast majority of cholera patients (>90%) void lytic vibriophage in addition to *V. cholerae*. Over a 5-year study of patients at the International Centre for Diarrhoeal Disease, Bangladesh (ICDDR,B), at least half of cholera patients harbored lytic vibriophage [29]. The ubiquity of lytic phage at the end of an outbreak suggests phage may play an important role in stopping an outbreak. This hypothesis is also supported by mathematical models [30], as well as epidemiological data that indicate household contacts of an index case that does not harbor lytic phage are at an increased risk of infection with *V. cholerae* [29].

In summary, a cholera outbreak is currently modeled as follows: An outbreak begins with the consumption of ABNC *V. cholerae* from the environment, is accelerated by hyperinfectious bacteria shed from patients, and is terminated by a rise in lytic phage. This model however does not provide a reason (selective pressure) for *V. cholerae* to leave the aquatic environment and go to the next host. Contrary to the current model regarding the importance of ABNC *V. cholerae* for transmission, we show that the loss of culturability is a negative selective pressure for transmission, and non-culturable cells are not the major contributors to infection. Instead we show here that culturable *V. cholerae* recently shed by patients are the major contributors to infection, and upon prolonged incubation in pond water, lytic phage and ABNC cells rise in the aquatic environment to cooperatively block transmission. In addition, transcriptional analysis suggests that bacteria quickly adjust to the stresses of the aquatic environment, and lytic phage have an undetectable influence on this adaptation. Despite this adaptation, rice-water stool *V. cholerae* rapidly become ABNC. In the absence of high-titer phage, our results support the model that recently shed hyperinfectious *V. cholerae* drive cholera outbreaks.

## Materials and Methods

### Bacterial strains and growth conditions

The strains used in this study are provided in Table S1. Strains were grown on Luria-Bertani (LB) agar or in LB broth with aeration at 37°C with streptomycin (SM) 100 µg/ml unless otherwise specified. SM sensitive strains were cultured on LB or a *Vibrio* spp. selective medium, TTGA [31]; the plating efficiency on TTGA and LB was equivalent (data not shown). The *in vitro* derived *V. cholerae* were prepared by growth for 4 h at 37°C with gentle rocking in M9 minimal medium (pH 9.0) supplemented with trace metals, vitamins (Gibco MEM Vitamins, Invitrogen), and 0.5% glycerol [32]; this medium is referred to as ‘M9 pH 9’.

### Pond microcosm system

Water was collected from a pond in central Dhaka each day of experimentation using a mechanical pump and intake hose system that collected water approximately 0.5 m below the water surface to avoid fluctuations in osmolarity due to rain water stratified at the top layer of the pond. This pond has historically cultured positive for *V. cholerae*; however in this study, *V. cholerae* and phage lytic for *V. cholerae* were below the limit of detection by standard methods [29] on the days of experimentation. Eighty liters of unfiltered pond water were transferred to a barrel lined with a pond-water washed autoclave bag, an aquarium bubbler was placed in the barrel to oxygenate the water as well as to avoid stratification, the barrels were positioned in an open shed shielded from direct sunlight but freely exposed to the outside air: water temp. 26–28°C, dissolved oxygen ≈6%, conductivity 260–300 µS/cm, total dissolved solutes 137.8 mg/l, salinity = 0.1 ppt, and pH 6.6–6.9. One liter of the water was centrifuged at 2,744 g at room temperature (RT) for 5 min, and filter sterilized through a 0.2 µm filter (FS pond water). This FS pond water was used to resuspend *in vitro* and *in vivo* derived *V. cholerae*, as well as for chemical analysis. After each experiment, bleach was added to each barrel to 0.5% and held for 24 hrs to sterilize the contents. Cultures on LB agar were taken to confirm complete sterilization before the water and bag were disposed. Inorganic chemical analysis on stool supernatant and pond water samples was performed by Dr. R. Auxier at the Center for Applied Isotope Studies (U. of Georgia, Athens, GA). Dr. A. Parastoo at the Complex Carbohydrate Research Center (U. of Georgia, Athens, GA) determined the major sugars in the FS pond water samples using mass spectrometry.

### Preparation of stool and in vitro derived *V. cholerae* for incubation in the pond microcosm

Stool samples were collected from adult patients (>15 yrs of age) with acute watery diarrhea and no prior treatment with antibiotics. The samples were examined by darkfield microcopy to confirm the presence of *V. cholerae* [29], and were included in the study if >95% of the cells were highly motile and vibrioid in shape. All samples were screened and found to be negative for ETEC, the ratio of *V. cholerae* to non-*V. cholerae* bacteria was determined, and the presence of lytic phage was assayed as previously described [29].

Stool samples meeting the inclusion criteria were clarified of mucus and debris by centrifugation at 988 g for 3 minutes at RT, and then *V. cholerae* were pelleted by 15 minutes of centrifugation at 26,892 g. Bacterial pellets were resuspended in an equal volume of FS pond water at a final concentration of approximately 1×10^8^ CFU/ml; alternatively, pellets were resuspended in RNAlater (Ambion, INC), flash frozen, and stored at −80°C for subsequent microarray analysis. Fifty ml aliquots of the resuspension were transferred to dialysis tubes with a 12 KDa cutoff (Fisher Scientific INC), and the tubes were immediately transferred to the pond microcosm described above. The tubes were kept just below the surface of the water, and bacteria and phage did not traverse the dialysis tubing (data not shown). The time from stool collection in the hospital to incubation in pond water was under 1 h. The collection of the rice-water stool from human subjects was reviewed and approved by both the Research Review Committee and Ethical Review Committee at the International Centre for Diarrhoeal Disease Research, Bangladesh, and by the Human Research Committee at the Massachusetts General Hospital.

*V. cholerae* were isolated by single colony purification from stool on either LB SM or TTGA media [31]. The *in vitro* derived *V. cholerae* were prepared as described above in M9 pH 9 at a final concentration of 1×10^8^ cfu/ml (approximately equivalent to the density in stool). After incubation at 37°C with gentle rocking for 4 h, the cells were then pelleted by centrifugation at 26,892 g for 15 min at RT, resuspended in an equal volume of FS pond water, transferred to dialysis tubes, and placed in the pond microcosm in a manner similar to the stool derived *V. cholerae* described above. Additionally, a portion of the pellets were stored in RNAlater as above for subsequent microarray analysis. At 5 and 24 h, the contents of the dialysis tubes (patient and *in vitro* derived) were transferred to sterile centrifuge tubes. For microarray analysis, the contents were centrifuged at 26,892 g for 15 min at RT, and the pellets were stored in RNAlater as above. At the 0, 5, and 24 h time points of collection for patient or *in vitro* derived cells for microarray analysis, paired samples were simultaneously taken for animal experiments described below.

### Infection experiments in the infant mouse model

The competitive index of *V. cholerae* pre-incubated in M9 pH 7, M9 pH 9, or rice-water stool supernatant was determined using 5 to 6-day-old Swiss Webster mice as described previously [22]. In brief, the O1 El Tor Inaba strain N16961 (LacZ^−^) of *V. cholerae* was grown overnight on LB agar with SM, and colonies were resuspended in LB broth. The cells were washed and incubated in M9 pH 7, M9 pH 9, or phage negative stool supernatant. During the incubation of the *in vitro* samples, stool samples from cholera patients were screened for *V. cholerae* and processed as described above. After the 1 h incubation of the *in vitro* grown strains, infant mice were inoculated intragastrically with 10^5^ CFU of a 1∶1 mixture of the paired LacZ^+^ stool *V. cholerae* and *in vitro* grown LacZ^−^ wild-type N16961 strain. At 24 h post inoculation, the small intestine was harvested and the homogenized contents were serially diluted and plated on LB SM, X-gal 40 µg/ml agar plates. After overnight incubation at 37°C, blue and white colonies were counted to determine the competitive index.

To study the infectivity of *V. cholerae* transferred to the pond microcosm, the ID_50_ was determined for both stool and *in vitro* derived *V. cholerae* after 0, 5, and 24 h of incubation in pond water. At 0 h, stool derived *V. cholerae* were prepared as described above and serially diluted in LB. The *in vitro* derived *V. cholerae* were prepared as described above with the 4 h preincubation at 37°C in M9 pH 9, and subsequent serial dilution in LB. Groups of 5–6 day-old Swiss Webster mice were then inoculated intragastrically with doses that ranged from approximately 1 to 10^5^ CFU per mouse. Mice were euthanized at 24 h post inoculation, and the small intestinal homogenates were plated as described above. Values of ≥1,000 CFU/mouse (limit of detection = 100 CFU) were recorded as positive for infection. A dose-response curve was made by plotting the fraction of infected mice against the log_10_ of the input *V. cholerae* cell count – either by CFU or direct counts. The ID_50_ was estimated from this curve by a standard nonlinear regression using the Hill Equation – the Hill slope was fixed at 1.0 when there were <3 data points between the values of 0.1 and 0.9 on the Y axis. The 95% confidence intervals for the ID_50_ (CI) and coefficient of determination (*R* ^2^) are provided. The ID_50_ for the stool and *in vitro* derived *V. cholerae* incubated in the pond microcosm was determined at 5 h and 24 h in the same manner.

The *in vivo* dynamic between *V. cholerae* and lytic phage was investigated by the co-infection of both the bacteria and lytic phage in ID_50_ experiments as described above. Lytic phage isolates were obtained in a pair-wise manner from the same patients that the *V. cholerae* isolates were obtained. Phage were isolated from stool supernatant by a standard plaque assay on a bacterial lawn made of the *V. cholerae* isolate from the same stool sample [33]. Phage were picked from 3 serial clear lytic plaques. *V. cholerae* were prepared for the animal studies by overnight growth and a 4 h incubation at 37°C in M9 pH 9 as described above. The *V. cholerae* isolated from a given patient and the paired lytic phage were combined for 8 min prior to infection at a phage::bacterium multiplicity of infection (MOI) that reflected what was observed in the rice-water stool and pond microcosm in this project: 0.001 to 5 PFU/CFU. The inocula were then serially diluted and groups of at least five infant mice were inoculated with doses that ranged from approximately 1 to 10^5^ CFU per mouse. The ID_50_ was calculated for each experiment as described above. At a given dose of bacteria and phage, the burden of infection was determined by calculating the median CFU/ml for each group of at least five mice. This was repeated for a total of 3 strains at all doses of bacteria and paired phage. The three medians were plotted individually, and the average of the three medians was also plotted. A Student's t-test was performed between the average for the no-phage control and each phage dose.

### Analysis of Cell Surface Polysaccharides

We investigated if colonization of infant mice by *V. cholerae* in the presence of lytic phage was because the bacteria had become resistant to the phage. One way that bacteria can become resistant to phage is by altering the phage receptor which is most commonly LPS for vibriophage [34],[35]. A basic test for putative LPS mutants is agglutination in LB [36]; this test lacks absolute specificity as other phenotypes can also cause agglutination such as expression of the toxin co-regulated pilus (TCP), but TCP is not expressed in LB [37]. We validated the agglutination assay with LPS extraction and gel electrophoresis of several putative LPS mutants identified by agglutination (below). We chose strain EN159 for this study because it is SM resistant. From each animal coinfected with EN159 (all doses) and the paired EN159 phage (all doses), eight isolates were colony purified (3×) and frozen for further evaluation of phage sensitivity. These isolates were grown in LB SM broth overnight and agglutination of the cells was assessed if the media clarified after 20 min of static incubation. The fraction of the 8 isolates from a given mouse that agglutinated was recorded as a fraction of isolates from a given mouse that were putative LPS mutants [36]. All isolates from mice infected with the highest dose of *V. cholerae* (1.5×10^5^ CFU/mouse) and all phage MOI's (0, 0.005, 0.1, and 2.0) were further tested for phage resistance by the standard plaque assay.

For validation of the agglutination assay, LPS was extracted from a total of five isolates from 5 different mice infected with the highest bacterial dose (1.5×10^5^ CFU/mouse) and at highest MOI (2.0). As a control, LPS was extracted from a total of five isolates from 5 different mice infected with the highest dose of bacteria (1.5×10^5^ CFU/mouse) and no phage. The input strain also served as an additional control. Cell surface polysaccharides from the eleven strains were isolated and analyzed as described recently [38],[39]. Briefly, Proteinase K-digested whole cell extracts were isolated according to Hitchcock and Brown [40] and analyzed by electrophoresis on 16.5% SDS-polyacrylamide gels. The complete synthesized LPS and the lipid A-core oligosaccharide precursor were visualized by silver staining [41].

### Microarray experiments

RNA was prepared from the samples collected at 0, 5, and 24 h of dialysis in pond water (described above). The frozen suspensions of bacteria in RNAlater (Qiagen) were thawed on ice, spun at 15,000 g for 20 min at 4°C, the supernatant was discarded, RNA was extracted from the pellet using the Qiagen (Valencia, CA) RNeasy Mini Kit, and DNA was removed using the Qiagen on-column RNase-Free DNase set. For qRT-PCR validation, complete DNA removal was achieved using the Ambion (Applied Biosystems/Ambion, Austin, TX) DNA-*free* DNase Treatment kit. Each RNA sample was spiked with an *in vitro* transcribed *Arabidopsis* RNA which served as a reference for color balancing during scanning; the control RNA was provided by the Pathogen Functional Genomics Resource Center (PFGRC) at the J. Craig Venter Institute (formerly TIGR). Labeling of cDNA was performed as described previously [42] with the exception that the reverse transcription reaction used Superscript III (Invitrogen, Carlsbad, CA) at a reaction temperature of 52°C for 1 h and 8 µg RNA. The cDNA from each reaction was split and labeled with either Cy3 or Cy5 (dye swapped). Unless indicated otherwise, at least 4 technical microarray replicates (2 dye swaps) were performed per biological replicate. There were two biological replicates for each condition: patient derived with phage, patient derived with no phage, and *in vitro* derived.

Microarrays were provided by PFGRC and consisted of glass slides with genes spotted in quadruplicate with 70 bp oligonucleotides for each of 3810 *V. cholerae* ORFs. Hybridizations were performed as described previously [42]. Microarrays were scanned with a Perkin-Elmer Scanner, and the raw data were analyzed using the Perkin-Elmer Scan Array Express, Imigene, and Spotfire software packages. Cy3 and Cy5 data from each slide were split into the relevant biological groupings as single channel data. All items with a raw intensity of less than 50 were assigned a minimum intensity value of 49.9 [43]. The complete data set was log_2_ transformed and normalized against all other scans by the 75^th^ percentile. The values for a given gene across all scans were then normalized by the z-score for that specific gene. The normalized data was then compared by ANOVA according to the relevant biologic grouping [44],[45],[46]. For ANOVA analysis between 6 groups, a Bonferroni correction was applied to account for bias due to multiple tests by dividing the desired level of significance (α = 0.01) by the total number of comparisons performed (22,860 = 3,810 genes with 6 comparisons) [44],[45],[46]. Therefore, the corrected false-positive rate was α = 4.4×10^−7^ which was rounded to α = 1.0×10^−7^; *P* values that fell below 1.0×10^−7^ were considered statistically significant. Cluster analysis was performed by Spotfire with the following metrics: clustered by Unweighted Pair-Group Method with Arithmetic mean (UPGMA), correlated by Pearson Product Momentum Correlation, and ordered by Input Rank. As an independent measure of similarity between biological groupings, Principal Component Analysis (PCA) was performed on all samples using Spotfire. After the ANOVA, all replicates were ungrouped, and the cluster analysis and PCA were performed in an unsupervised fashion with respect to the technical replicates and biological groupings. Fold-changes between two biological groupings were calculated using distinction calculations performed by Spotfire, and fold-changes with *P* values<6.6×10^−6^ (Bonferroni corrected) were considered significant. Microarray data are available in the supplemental material (Tables S3, S4, S5, S6, S7, S8, S9 and S10).

### Validation of microarray by quantitative qRT-PCR

There was sufficient sample to obtain cDNA template from three phage positive patients (EN159, EN182, EN191), and three phage negative patients (EN124, EN150, EN174). RNA was isolated as described above, and qRT-PCR was performed as previously described [13]. In brief, cDNA was synthesized from 1 µg of RNA using the SuperScript II First Strand Synthesis System for qPCR (Invitrogen Inc.). The qRT-PCR experiments were performed with iQ SYBR Green supermix (Biorad). Each reaction contained 200 nM primers, approximately 10 ng of the template, and the ROX reference dye. All primer pairs (Table S1) amplified the target with efficiencies of 92% or greater (data not shown). The mean cycle threshold for the test transcript was normalized to the reference transcript *sanA* [47] and *argS*. The reference *argS* was chosen because no expression changes were detected in this microarray project as well as all publicly available *V. cholerae* microarray databases. Values >1 indicate that the transcript is in higher concentration than the reference.

## Results

### Sample collection from cholera patients at the ICDDR,B

This project focuses on rice-water stool samples collected from three patients (EN159, EN182, and EN191) who harbored lytic vibriophage for *V. cholerae*, and the respective phage and *V. cholerae* isolates from these three patients. In addition, rice-water stool was collected from three patients who did not harbor lytic vibriophage (EN124, EN150, and EN174), and *V. cholerae* was isolated from each of these patients. Therefore, the biological replicates for each arm of the study were three unless stated otherwise; sufficient numbers of infant mice for ID_50_ testing were available only for the three patient samples that harbored phage. At the time of collection, all patients were severely dehydrated as defined by the World Health Organization [48].

### Chemical analysis of pond water

**Figure 1:**
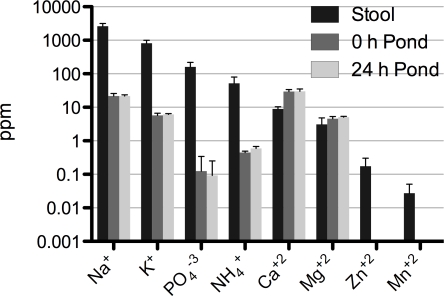
Change in concentration of biologically relevant elements upon passage from patients to the pond environment. Data represent the average and standard deviation for three independent patient rice-water stool samples (EN159, EN182, EN191) and the pond water samples collected on the respective days of experimentation. ‘0 h Pond’ (dark grey) and ‘24 h Pond’ (light grey) represent filter sterilized pond water from the inside of dialysis bags used to incubate *V. cholerae*, respectively. Zn and Mn were below the level of detection in the pond. In addition, Al, B, Ba, Cd, Co, Cu, Fe, Mo, Ni and Pb were below the level of detection; ppm = parts per million.

As *V. cholerae* passes from the patient into pond there is dramatic shift in osmolarity and in the concentrations of inorganic nutrients and carbon sources. Some of these factors are depicted in Fig. 1. NaCl and KCl are major contributors to osmolarity and both have a decline from 2,600 to 22 ppm (120-fold) and 820 to 6 ppm (140-fold) between the rice-water stool and pond supernatant, respectively. The conductivity difference between the rice-water stool (as well as LB broth) and pond water is approximately a 50-fold decline. Phosphate and fixed nitrogen are typically limiting inorganic nutrients in fresh water ponds. Phosphate and fixed nitrogen (NH_4_^+^) decline from 160 to 0.1 ppm (1,600-fold) and 52 to 0.5 ppm (104-fold), respectively. *V. cholerae* was placed in filtered pond water and then dialyzed in 12 KDa tubing with live pond water. Therefore, carbon sources such as large polymers like chitinous exoskeletons would not be present in the dialysis bags. Carbon sources detected were rhamnose (29 Mol.%; 16 nM), fucose (20% Mol.%; 11 nM), glucose (2.7 Mol.%; 1 nM), and unidentified sugars (48.9 Mol.%). This chemistry collectively framed many of the physiological events that occurred as *V. cholerae* adapted to the aquatic system. This adaptation and pond microcosm system is not necessarily specific to Bangladesh as the chemical composition shown herein is comparable to pond water used in transition studies with pond water obtained in Boston, MA [13].

### Exponential drop of culturability in a pond microcosm

**Figure 2:**
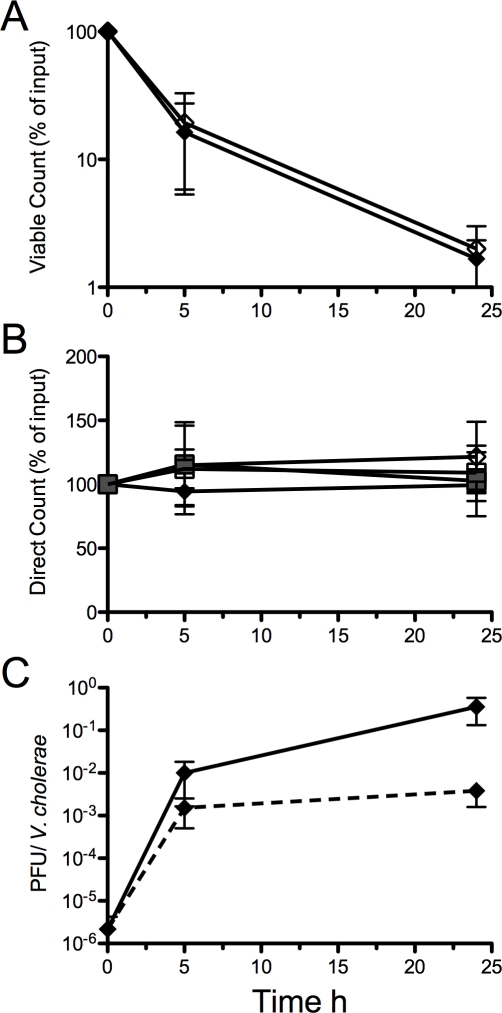
*V. cholerae* and phage counts during a 24 h incubation in pond water. A. The CFU decline between the patient derived (solid diamonds, N = 3) and *in vitro* derived (clear diamonds, N = 3) *V. cholerae* is similar. All patient samples had phage (EN159, EN182, EN191; N = 3). B. Direct counts (DC) do not change over 24 h. DC for *V. cholerae* from patient samples with phage (solid diamond, N = 3) and with no phage (shaded squares, N = 3). The *in vitro* grown strains isolated from phage positive stools (N = 3) or from phage negative stools (N = 3) are clear diamonds and clear squares, respectively. C. Phage bloom at 5 h (N = 2). The ratio of plaque forming units (PFU) to *V. cholerae* is plotted in two ways: PFU to CFU ratio (solid line); PFU to DC ratio (dashed line).

The culturability of *V. cholerae* transferred to the pond microcosm was monitored by culture and direct microscopy counts. We define the non-culturable cells as ‘active but non-culturable’ (ABNC) because there were clear transcriptional changes between 5 and 24 h detected by both microarray and qRT-PCR analysis (below). Thus, our measure of ‘active’ was global transcriptional change. Culturability was rapidly lost upon transfer to the pond microcosm at 5 and 24 h with declines of 63% (SD+/−16%) and 98% (SD+/−1.0%), respectively (Fig. 2A). The *V. cholerae* isolates from the respective patients were grown *in vitro* (M9 pH 9) and transferred to the pond microcosm; the declines in culturability in the pond microcosm were similar for the *in vitro* derived samples compared to the patient derived samples (Fig. 2A). Despite the drop in culturable cells, the total cell numbers remained constant by direct counts (Fig. 2B) for all sample types; the cell number was also constant for phage negative patient samples and the paired *in vitro* grown strains (Fig. 2B). The culture counts are not available for the phage negative patient samples because two isolates were unexpectedly SM sensitive. The plating efficiency of starting cultures neared 100%. For example, the average concentration of *V. cholerae* from patients (EN159, EN182, EN191) at 0 h by culture counts and direct counts was 1.0×10^8^ CFU/ml (+/−1.1×10^8^ CFU/ml) and 1.65×10^8^ CFU/ml (+/−0.35×10^8^ CFU/ml), respectively.

### Lytic phage from patients bloom in the pond microcosm

**Figure 3:**
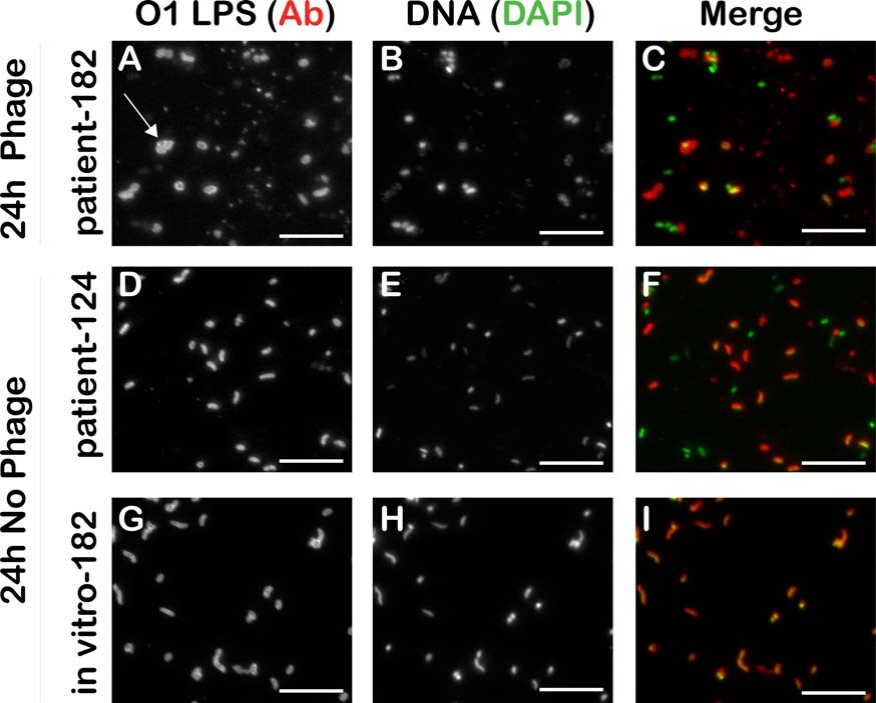
Phage positive patient derived *V. cholerae* have irregular shape at 24 h (arrow). Representative images of a lytic phage positive patient sample (EN182; A, B, C) and phage negative patient sample (EN124; D, E, F) incubated in pond water over 24 h. The isogenic strain EN182 was isolated away from the phage, grown *in vitro* and incubated for 24 h in the pond water (G, H, I). *V. cholerae* are labeled with a FITC conjugated monoclonal Ab to O1 LPS (left) and nucleic acid is labeled with DAPI (middle) which indicates non-*V. cholerae*; merge is shown on right. Data are representative of 3 independent experiments (data not shown). *V. cholerae* from the 0 h and 5 h time points for all sample types were indistinguishable by morphology and represented ‘F’ and ‘I’. Scale bar = 10 µm.

The PFU titer was monitored at 0, 5 and 24 h in the pond microcosm (Fig. 2C). At 0 h, the average ratio of phage to *V. cholerae* for all three patient stools was 2.2×10^−6^ (SD+/−3.5×10^−6^ ). At 5 h, this ratio increased by 4 orders of magnitude to 1.0×10^−2^ (SD+/−1.2×10^−2^) by culture counts, or 3 orders of magnitude to 1.5×10^−3^ (SD+/−1.3×10^−3^) by direct counts. At 24 h, this ratio increased an additional 2 orders of magnitude to 4.0×10^−1^ (SD+/−3.9×10^−1^) by culture counts, but remained steady at 3.8×10^−3^ (+/−3.2×10^−3^) by direct counts. From 5 to 24 h, this ratio changed because the culturable counts decreased 14-fold. These findings are supported by micrographs that illustrate altered morphology of *V. cholerae* only in the patient derived samples from phage positive patients (Fig. 3A). Lytic and lysogenic vibriophage have been previously characterized from patients [27],[33],[35],[49],[50],[51],[52]; our phage isolates are consistent in terms of the tropism of those lytic phage previously published [27] because our phage had specificity for the Inaba or Ogawa serotype of the O1 El Tor *V. cholerae* biotype, and the phage were unable to form plaques on O139 *V. cholerae* (data not shown). These data indicate the phage receptor may be O1 LPS as has been demonstrated previously [34],[35]. Support for this hypothesis is the generation of LPS mutants in the presence of lytic phage (presented below).

### Hyperinfectivity is maintained for at least 5 h of dialysis in pond water

**Figure 4:**
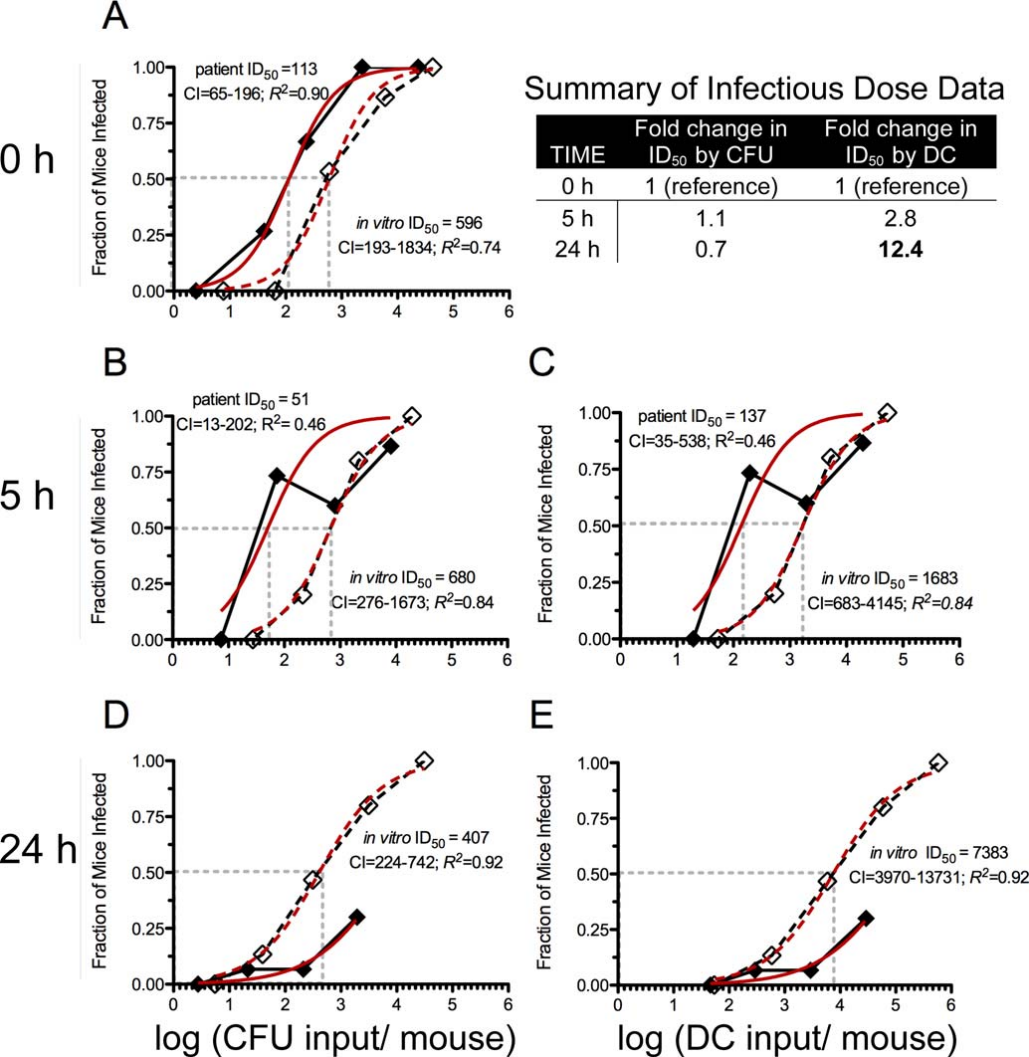
ID_50_ experiments of *V. cholerae* incubated in pond water. *V. cholerae* were incubated in pond water for 0 (A), 5 (B, C) and 24 h (D, E). Each symbol represents the average of the fraction of infant mice infected with *V. cholerae* from three phage positive patient samples (solid diamond/solid line), or the same three isogenic strains from *in vitro* culture (clear diamond/dashed line). The data are plotted on the X axis by the log of the culturable inputs (CFU) on the left or direct count inputs (DC) on the right; the log (ID_50_) is depicted with the grey dashed vertical line. The inset table summarizes the fold changes in the ID_50_ over 24 h for the *in vitro* derived samples. CI = 95% confidence interval for the ID_50_ determined from the fitted curve (red) modeled with the Hill Equation. Plating efficiency at 0 h neared 100% (see text). Each symbol represents the average of the 3 biological replicates (EN159, EN182, EN191) which pools data from at least 15 mice total.

The ID_50_ for *V. cholerae* freshly shed from the patients (113 CFU; 95% confidence interval [CI] = 65–196 CFU) was lower compared to the *in vitro* grown reference (596 CFU; 95% CI = 193–1834 CFU; Fig. 4A). Hyperinfectivity was also observed after 5 h of dialysis between the patient (51 CFU; 95% CI = 13–202 CFU) and *in vitro* culture (680 CFU; 95% CI = 276–1673 CFU; Fig. 4B). These findings are consistent with competition experiments previously published that suggest *V. cholerae* maintains hyperinfectivity for at least 5 h after exit from the patient [23]. We tested if hyperinfectivity could be induced by the medium alone (stool-supernatant), and we found that hyperinfectivity could not be induced *in vitro* by incubation in stool supernatant (pH 9) or minimal media (M9 pH 9) (Fig. S1). Unique to the present study was that the single strain infection experiments revealed that the fraction of mice infected with high doses of patient derived *V. cholerae* was reduced at 5 h and 24 h compared to the *in vitro* reference (Fig. 4B–E). Indeed, the ID_50_ was not able to be calculated for the patient derived samples at 24 h because less than 50% of the animals were infected (Fig. 4D–E). The 24 h time point corresponds with the point when the titer of PFU was highest and the titer of culturable cells was lowest (Fig. 2); note again that the no phage control for this experiment are *in vitro* derived cells. We hypothesized, and show below, that the incomplete colonization observed is due to the presence of lytic phage in the inocula.

### Culturable *V. cholerae* are the major contributors to infection

We wanted to investigate the relevance of the ABNC state to the transmission of *V. cholerae*. To do this we tracked the ID_50_ over time by both culturable counts and direct counts. We focus here on the ID_50_ data from the *in vitro* derived *V. cholerae* because the phage positive patient derived samples failed to fully colonize at 5 and 24 h. In the context of the pond system, the total cell counts remained constant but the proportion of culturable cells decreased over time. We tested three competing hypotheses: (i) If culturable cells are equally infectious as non-culturable cells, then the ID_50_ by total cell counts will be constant as the percent of culturable cells decreases. (ii) If culturable cells are more infectious than non-culturable cells, then the ID_50_ by total cell counts will increase as the percent of culturable cells decreases. (iii) If culturable cells are less infectious than non-culturable cells, then the ID_50_ by total cell counts will decrease as the percent of culturable cells decreases. As mentioned above, the culture cell counts fell from 100% to 27% to 3% at 0, 5 and 24 h during the experiment. The corresponding ID_50_ by culturable counts remained constant as the culturable counts decreased at 0, 5 and 24 h (Fig. 4A, B, D). However, the ID_50_ by total cell counts rose from 596 (95% CI = 193–1834) to 1683 (95% CI = 683–4145) to 7383 (95% CI = 3970–13731) as the culturable counts decreased at 0, 5 and 24 h (Inset table in Fig. 4). Therefore, hypothesis (ii) appears to be correct that culturable cells are more infectious than non-culturable cells, and thus, the major contributors to infection are culturable *V. cholerae*.

### Coinfection of lytic phage and *V. cholerae* alters the burden of infection

**Figure 5:**
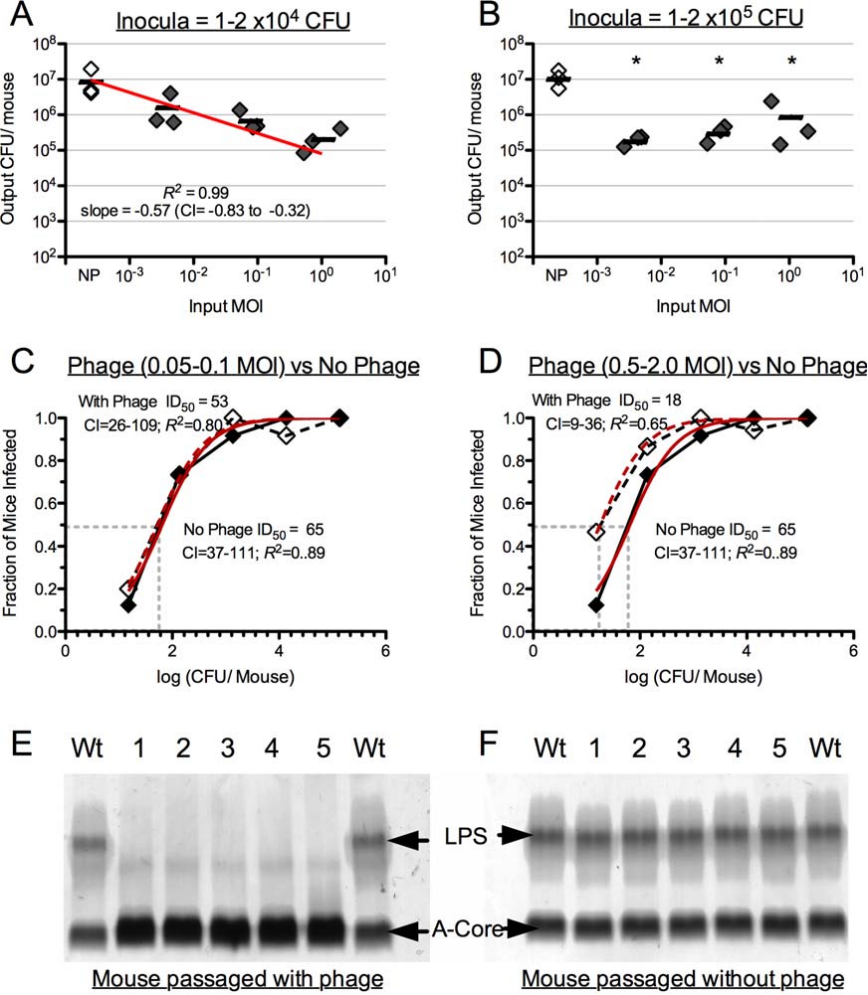
Coinfection of infant mice with *V. cholerae* and lytic vibriophage. A. Average colonization (bar) of mice inoculated with *in vitro* derived strains (EN159 EN182, EN191) co-infected with the paired phage isolate (solid diamonds). The bacterial dose is constant (1–2×10^4^ CFU/mouse) and the phage dose is variable ranging from a MOI of 10^−3^ to 1.0. NP = no phage control (clear diamonds). Each diamond represents the median of 5 technical replicates (mice) for a given biological replicate (EN159, EN182, EN191). CI = 95% confidence interval for the slope of the linear line. B. Same as ‘A’ except a constant bacterial dose of 1–2×10^5^ CFU/mouse. *Significant difference compared to the no phage control (Student's *t*-Test; *P*<0.05). C. ID_50_ plotted by the log(CFU/Mouse) against the fraction of mice infected. Inoculation with a constant phage dose (MOI = 0.05–0.1; solid diamond/line) and a variable dose of *in vitro* derived *V. cholerae*. No phage control = clear diamonds/dashed line; CI = 95% confidence interval for the ID_50_ calculated from the fitted curve (red) modeled with the Hill Equation. Each symbol represents the average of 3 biological replicates (≥5 mice per biological replicate). D. Same as ‘C’ except with a constant phage dose of MOI = 0.5–2.0. E. Analysis of LPS isolated from wild-type EN159 (Wt), or mouse passaged EN159 (1–5) isolated from mice infected at 1.5×10^5^ CFU/mouse as in ‘B’ and an MOI of 2.0. Upper arrow indicates fully synthesized LPS with attached O antigen, lower arrow indicates the lipid A-core oligosaccharide precursor. F. Same as ‘E’ except mouse passaged isolates (1–5) were in the absence of phage (NP).

Because lytic phage are present in aquatic reservoirs and in at least half of cholera stool samples, we wanted to determine the relevance of lytic phage to the transmission of *V. cholerae*. We hypothesized that the reduction in colonization at 5 and 24 h for the patient derived samples (Fig. 4) was caused by the bloom of lytic phage because no such reduction was observed for phage minus *in vitro* derived *V. cholerae*. We tested this hypothesis by coinfecting infant mice with *V. cholerae* isolated from the three phage positive patients (EN159, EN182, EN191), and the paired lytic phage isolate from each patient (described above). The inocula were made by mixing bacteria and phage at various MOI's that were relevant to those observed in rice-water stools and after incubation in the pond microcosm (Fig. 2C). A linear dose response (*R* ^2^ = 0.99; slope = −0.57; 95% CI = −0.83 to −0.32; Fig. 5A) was observed in the infant mice inoculated with a constant bacterial dose (1–2×10^4^ CFU/mouse) and variable phage dose (1.0×10^−3^−2.0 MOI). In contrast, at a high dose of *V. cholerae* (1–2×10^5^ CFU/per mouse) there was a significant reduction in colonization at all MOI tested (Fig. 5B).

The coinfection experiments were also performed as ID_50_ experiments with variable concentrations of *V. cholerae* and 4 constant doses of phage (MOI = 0, 0.005–0.1, 0.05–0.1, 0.5–2.0). There was no difference in the ID_50_ between the no phage control and animals coinfected with phage at an MOI of 0.05–0.1 (Fig. 5C) and 0.005–0.01 (data not shown). However, the ID_50_ in mice co-infected with phage at a MOI of 0.5–2.0 (18 CFU; 95% CI = 9–36) was significantly lower than the no phage control (65 CFU; 95% CI = 37–111 CFU). These experiments support the hypothesis that phage can limit infection at doses of *V. cholerae* greater than 10^3^ CFU. However at high phage MOI and low doses of *in vitro* grown *V. cholerae*, the phage may have an unexpected positive impact on the ID_50_ (Fig. 5D). EN159 isolates from experiments in which the phage may have had a positive impact on infectivity (MOI of 2.0; 100–200 CFU) were found to be phage sensitive and not LPS mutants (data not shown). In competition in the infant mouse model, these isolates competed 1∶1 with the input strain suggesting there was no gain of function from prior co-culture with the phage (data not shown).

Although the phage reduced the burden of *V. cholerae* infection, complete clearance of the bacteria was not observed, as had occurred in the pond microcosm (Fig. 4E). To investigate a reason behind this we closely examined isolates from the EN159 coinfection studies because EN159 is SM resistant. 40–70% of isolates from mice coinfected with EN159 *V. cholerae* at a dose of 1–2×10^5^ CFU/per mouse agglutinated in LB independent of the phage dose – a phenotype consistent with LPS mutants [36]. No isolates from mice coinfected with *V. cholerae* at a dose of less than or equal to 1–2×10^3^ CFU/per mouse agglutinated. To confirm that agglutination was indicative of LPS mutations in our system we analyzed LPS from several isolates. The LPS from a total of five isolates from five different mice infected with the EN159 *V. cholerae* and phage (MOI = 1.0) was compared to the LPS from a total of five isolates from different mice infected with EN159 *V. cholerae* and no phage. All five mouse passaged isolates from coinfection experiments with phage were resistant to the phage and agglutinated, and the five mouse passaged isolates without phage were sensitive to the phage and did not agglutinate. These phage sensitive colonies demonstrated a wild-type LPS with the typical two band pattern consisting of the lipid A-core oligosaccharide precursor as the lower band and the complete LPS with attached O antigen as the upper band (Fig. 5F). In contrast, phage resistant colonies exhibited an O antigen deficient phenotype (Fig. 5E). We were concerned about lysogeny among phage resistant colonies. Phage sensitive *V. cholerae* were infected with phage *in vitro* and subsequent treatment of phage resistant isolates with and without mitomycin-C [53] yielded no phage; this experiment was repeated for all strains and phage in this study. These data suggest there was no lysogeny. However, experiments with additional stresses (osmotic shock, UV, etc.) and phage isolates genetically marked with an antibiotic resistance marker would be required to definitively show the absence of lysogeny. Taken together, these data indicate that at a high dose of *V. cholerae*, spontaneous LPS mutants will dominate during *in vivo* colonization in the presence of lytic phage.

### Global transcriptional analysis of ABNC *V. cholerae* in the aquatic environment

**Figure 6:**
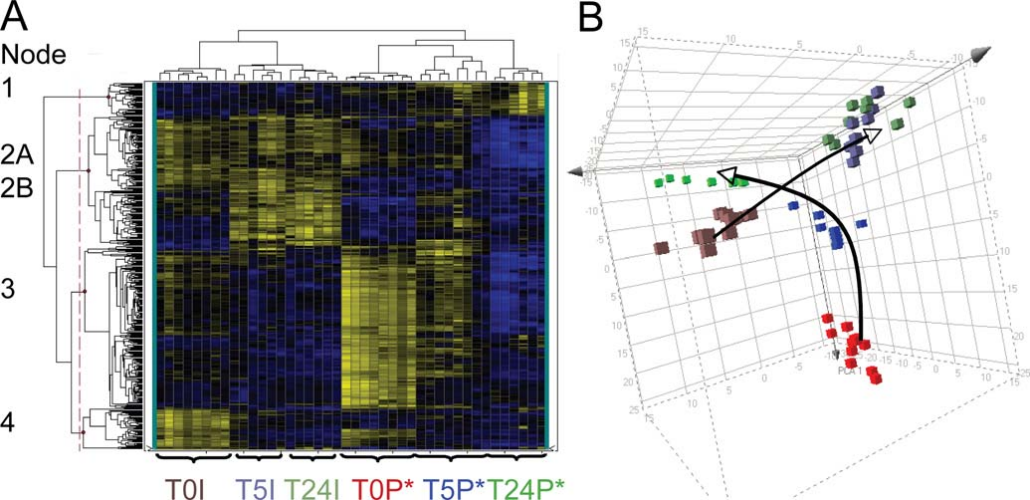
Transcriptional profiles of patient derived and *in vitro* derived *V. cholerae* incubated in a pond microcosm. A. Heat map of 435 genes that are differentially regulated (ANOVA *P*<1×10^−7^; N = 2) in at least one of the following six conditions: *V. cholerae* from patients (T0P*, T5P*, T24P*) or *in vitro* culture (T0I, T5I, or T24I) incubated for 0, 5, or 24 h; the patient samples harbored phage*. Yellow and blue represent induced (max = 46-fold) and repressed (max = 20-fold) genes, respectively. The sample labels at the bottom are color-coded to match the right panel. The thin vertical dotted line breaks the genes into four major groups (nodes) provided in the supplement from top to bottom as Nodes 1-4 (Tables S3, S4, S5, S6 and S7). Node two is subdivided into 2A (upper; Table S4) and 2B (lower; Table S5). B. Principal Component Analysis (PCA) of the 435 gene expression values in ‘A’. The arrows indicate the ‘transcriptional movement’ from 0 to 5 to 24 h for the patient and *in vitro* derived *V. cholerae*.

**Figure 7:**
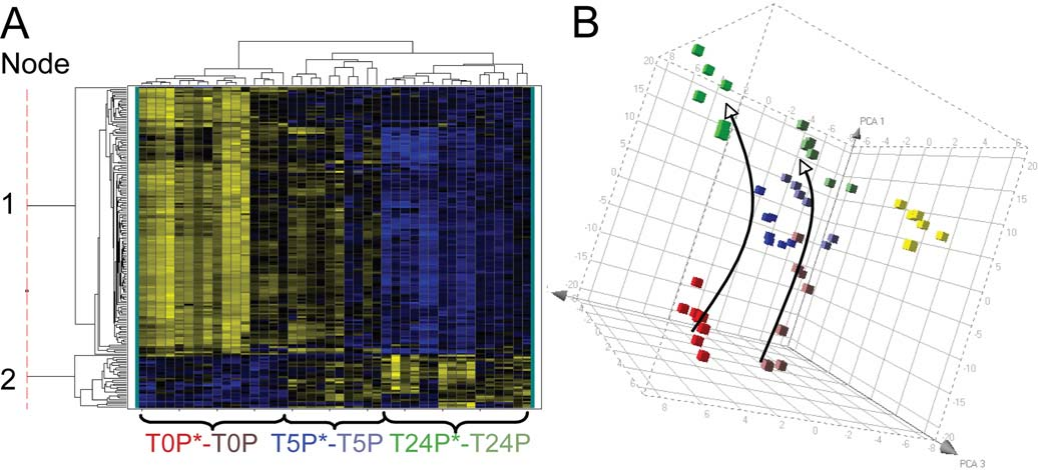
Transcriptional profiles of patient derived *V. cholerae* with and without phage incubated in a pond microcosm. A. Shown is a heat map of the subset of transcripts that are differentially regulated (ANOVA *P*<1×10^−7^; 182 genes) in at least one of the following six conditions: *V. cholerae* from phage positive patients (T0P*, T5P*, T24P*) or phage negative patients (T0P, T5P, or T24P) incubated for 0, 5, or 24 h. Yellow and blue represent induced (max = 46-fold) and repressed (max = 16-fold) genes, respectively. The sample labels at the bottom are color-coded to match the right panel; one T5P sample clustered with T5P* (as shown) and one T5P clustered with the T24 samples (not individually labeled). The thin vertical dotted line breaks the genes into two major groups (nodes) provided in the supplement as Node 1 (upper group; Table S8) and Node 2 (lower group; Table S9). B. Principal Component Analysis (PCA) of the 182 gene expression values in ‘A’. The arrows indicate the ‘transcriptional movement’ from 0 to 5 to 24 h. Biological Replicates = 2. Yellow cubes are the T24I *in vitro* derived samples depicted in Fig. 6B as a reference.

Because ABNC *V. cholerae* have low infectivity, yet represent the predominant state of the bacteria after 5 h of incubation in the pond microcosm, we measured possible transcriptional changes during this transition. The goal was to ascertain whether the bacteria were adapting to the nutrient poor conditions in a manner dependent or independent of their source of origin (patient or *in vitro*). Samples for the microarray fell into six biological groups: patient derived samples (EN159, EN182) incubated in the pond microcosm for 0, 5 and 24 h (designated T0P*, T5P* and T24P*, respectively; Fig. 6A) and the paired *in vitro* derived isolates incubated in the pond for 0, 5 and 24 h (designated T0I, T5I and T24I, respectively; Fig. 6A). Both patient (EN159, EN182) samples harbored phage, which is indicated with an asterisk. Two additional patient samples that did not harbor phage (EN124, EN150) were included as controls for transcriptional changes induced by phage (designated T0P, T5P and T24P, respectively; Fig. 7A). The patient samples EN174 and EN191 and *in vitro* samples EN124 and EN150 were excluded because of insufficient material for microarray analysis. The qRT-PCR validation is provided in Table S2. Cluster analyses in Fig. 6 and Fig. 7 isolated key expression patterns; genes within these groupings are described by biological function in Tables S3, S4, S5, S6, S7, S8, S9 and S10. A complete list of all fold changes is available in Table S10.

#### The transcriptional profile of patient and *in vitro* derived *V. cholerae* did not converge during transition to the ABNC state

The transcriptomes at each time point (0, 5 and 24 h) were highly concordant within each of the various biological groups (Fig. 6A and Fig. 7A). ANOVA analysis was used to select a population of 435 genes (*P*<1.0×10^−7^) that were differentially regulated in at least one of the six groupings stated above. These 435 genes are represented by the heat map and cluster analysis in Fig. 6A. Each technical replicate clustered into the appropriate biological grouping. At no time point did the transcriptome of the patient and *in vitro* derived samples converge on the global level. These results were cross-validated by independent analysis using principal component analysis (PCA) (Fig. 6B).

#### Transcriptional adjustment was rapid

The cluster analysis and PCA in Fig. 6 group the 5 and 24 h *in vitro* derived samples together. This suggests that the majority of the transcriptional adaptation to the pond occurs by 5 h. We explored this observation by performing cluster analysis between the 0, 5 and 24 h time points for each sample individually. ANOVA analysis was used to select a population of approximately 300 genes (*P*<1.0×10^−7^) that were differentially regulated between the 0, 5 and 24 time points. Five of the six cluster analyses demonstrated rapid adjustment to the pond as indicated by the 5 and 24 time points grouping together (data not shown) which is consistent with Fig. 6A.

#### The absence of convergence was not caused by phage

We tested if phage were responsible for the lack of convergence between the patient and *in vitro* derived samples by comparing phage positive (EN159, EN182) and negative (EN124, EN150) patient samples. A new ANOVA analysis was used to select a population of 182 genes (*P*<1.0×10^−7^) that were differentially regulated in at least one of the six groupings: T0P*, T0P, T5P*, T5P, T24P* and T24P. These 182 genes are represented by the cluster analysis and PCA in Fig. 7. The cluster analysis grouped the 0 h samples together independent of whether the samples harbored phage. The 24 h samples also clustered together independent of whether they harbored phage. One of the 5 h phage negative patient samples (T5P) clustered with the 24 h samples, and the second 5 h sample (T5P) clustered in a unique clade with both the 5 h phage positive patient samples (T5P*). The similarity between the patient derived samples suggests that phage had a limited influence on the transcriptome. A second analysis was performed on only the 435 genes depicted in Fig. 6. The *in vitro* specific transcripts were subtracted, and the remaining genes were analyzed by cluster analysis and PCA for the patient derived samples with and without phage. Again, there was no difference in profiles between the two types of patient derived samples (data not shown). One question is how the microarray data can be so similar between phage positive and negative samples at 24 h despite gross differences in cellular morphology (Fig. 3). One explanation is that the majority of the transcriptional changes occur by 5 h which is supported by the cluster analysis presented above. This explanation requires the RNA to remain intact to some degree from 5 to 24 h.

### Regulon specific analysis of ABNC *V. cholerae* in the aquatic environment

#### De-repression of virulence regulons in the pond

The microarray findings are consistent with previously published microarray studies that demonstrate at the point of exit from the human host there is a profound repression of virulence genes, and their relevant activators, that includes the toxin co-regulated pilus (TCP), cholera toxin (CT), and chemotaxis regulons relative to the *in vitro* derived reference [23],[47]. For example, cholera toxin genes (*ctxA* and *ctxB*) have divergent expression at 0 and 5 h but convergent expression at 24 h relative to the *in vitro* derived reference (Table S10). The repression of chemotaxis is hypothesized to be a contributing factor to the hyperinfectivity phenotype [22], [54]. By microarray, all three chemotaxis operons (#1 VC1394-1406, #2 VC2059-20-65, #3 VCA1088-1096) were repressed between rice-water stool *V. cholerae* and the *in vitro* derived reference. qRT-PCR analysis is consistent with these findings showing repression of approximately 2-fold and 4-fold for *cheW*-1 (operon #2) and *cheY*-4 (operon #3) with respect to the *in vitro* reference, respectively (Table S2). Expression rises approximately 4-fold and 8-fold for *cheW-1* and *cheY*-4 by 5 h, respectively by qRT-PCR (Table S2). The mechanism for the repression of chemotaxis genes in rice-water stool and de-repression in the pond remains unknown.

#### Adaptation to nutrient limitation

Transition from rice-water stool into the ABNC state in the aquatic environment is a process of adaptation to nutrient limitation (Fig. 1). There was commonality of expression profiles for genes related to phosphate limitation independent of where the sample was derived from (patient or *in vitro*). For example, *phoB* is part of the *pho* regulon that senses and responds to phosphate limitation [55],[56]. By qRT-PCR, *phoB* is induced approximately 5-fold and 39-fold in the first 5 h for patient and *in vitro* derived samples, respectively (Table S2). These data are consistent with the array data, but the absolute numbers are different because the microarray data are compressed. Fixed nitrogen limitation is a second nutrient of limited availability. Ammonia is incorporated to make glutamine by the action of GlnA (Glutamine Synthetase) and GlnB [57]. *V. cholerae* has two paralogs of *glnB*: *glnB*-1 and *glnB*-2. By qRT-PCR, *glnB-1* is induced in the patient derived samples by 5-fold in the first 5 h; the *in vitro* derived samples are already induced in the M9 pH 9.0 and decline in expression levels by 3-fold over the 24 h (Table S2). The qRT-PCR values are again consistent with the microarray values (Table S10). These data support a model for immediate adaptation to nutrient limitation coincident with entry into the ABNC state in the aquatic environment.

#### Suppression of protein synthesis

A second level of adaptation to the aquatic environment is at the level of global protein synthesis. In Fig. 6A, clusters of genes fall into 4 major groups (nodes) based on 4 expression profiles. Node 3 contains 174 genes that are strongly expressed in patients as well as *in vitro* and rapidly turn off in the pond. 34% of these genes (60 total) are involved in protein synthesis, and 14% (14 total) are involved in energy metabolism (Table S6). Many of the protein synthesis genes are contained within the VC2599-VC2570 ribosomal protein gene locus. This cluster was identified previously as a locus induced in patients [23],[42] and repressed in stationary phase compared to log phase [47]. The ribosomal gene *rplC* (VC2596) is near the beginning of the operon and is induced 2-fold by qRT-PCR (Table S2), compared to the *in vitro* reference at 0 h (Table S2). By microarray, 29 out of 30 genes at this locus decrease expression between 2-fold to 310-fold over 24 h (Table S10). This decline in the expression of ribosomal proteins suggests a general decrease in the capacity for protein synthesis as the cells enter the ABNC state.

#### RpoS as a candidate regulator of environmental adaptation

One component of the regulation of the ribosomal protein locus VC2599-VC2570 is the stationary phase alternative sigma factor RpoS [47]. RpoS has been determined to play a role in virulence in the infant mouse model [58] as well as regulate the mucosal escape response in the rabbit ileal loop model [47]. A microarray of an *in vitro* grown *rpoS* mutant compared to wild-type *V. cholerae* showed that the ribosomal protein locus VC2599-VC2570 was repressed in the mutant strain. This suggests that RpoS positively regulates VC2599-VC2570 [47]. However in our microarray, the VC2599-VC2570 locus is repressed in the pond while *rpoS* is simultaneously induced at least two-fold (Table S2). The regulation of VC2599-VC2570 is therefore likely complicated by additional unknown repressive factors that undermine the positive control by RpoS. Nevertheless, at the global level, RpoS regulated genes represented 28% of the genes depicted in Fig. 6 (123/435 genes) and Fig. 7 (51/182 genes). In comparison, RpoS regulates 11% of the transcriptome in stationary phase in LB (418/3810 genes) [47]. The difference between 28% and 11% is significantly different (*P*<0.01; Pearson's chi-square test), and the increased representation of RpoS regulated genes in the pond is therefore not due to chance alone. Future studies of *rpoS* mutants in pond water may aid in determining the role *rpoS* plays in the entry of *V. cholerae* into the ABNC state.

## Discussion

This project was designed to concurrently test three critical factors for their relevance to the transmission of cholera. The patient to pond microcosm system allowed us to evaluate (i) the infectivity of *V. cholerae* as the cells enter into (ii) the ABNC state in (iii) the presence or absence of lytic vibriophage. The ID_50_ data suggest that the major contributors to infection are culturable *V. cholerae*. Phage did not affect colonization immediately after passage from the patients because the PFU titer was likely too low. However, *V. cholerae* failed to colonize the animals after 24 h of adaptation to pond water – the point when the PFU titer and ABNC cells were highest. Taken together, these data challenge the concept that the aquatic environment is an amenable refuge for *V. cholerae* during transit between human hosts.

The entry into the ABNC state has been challenging to standardize experimentally because it is difficult to sufficiently dilute the cells to the point that they do not self fertilize key nutrients, and at the same time maintain a high enough cell density for tractable experimentation. These problems were overcome by using dialysis tubes containing *V. cholerae* in suspension in a large volume (80 L) of live pond water. In this system, culturability reproducibly fell by approximately 60% and 98% by 5 and 24 h, respectively, independent of the origin of the bacteria. Defining the physiologic state of cells that do not culture has been controversial. Herein we limit our work to two populations: cells that culture and those that do not culture. We do not differentiate within the population of non-culturable cells that may contain a subpopulation of dead cells. That said, the non-culturable cells are likely to be alive for several reasons: propidium iodide staining for intact membranes indicated the majority of cells in all arms of the study at 24 h had intact membranes (data not shown). Secondly, the RNA yield was similar between 0 and 24 h despite the 98% loss in culturable cells. Finally, the microarray data at 24 h showed continued adaptation in the pond. For example, genes involved with adaptation to low phosphate and fixed nitrogen were induced [59],[60]. Tests for metabolic activity were not performed on all samples. Instead, we define ‘active’ in the context of this project as the capacity for transcriptional change despite a lack of culturability.

Having defined the proportion of non-culturable cells at 24 h, we tested the relevance of ABNC cells to infection. In the context of the pond microcosm, the total cell counts remained constant but the proportion of culturable cells decreased over time. We tested several hypotheses to determine the role of non-culturable cells in transmission. One hypothesis stated that if culturable cells are more infectious than non-culturable cells, then the ID_50_ calculated by total cell counts will increase as the percent of culturable cells decreases. The data revealed that the ID_50_ by total cell counts rose as the culturable counts decreased at 0, 5 and 24 h. Therefore, these data suggest that culturable *V. cholerae* were the major contributors to infection. Previous studies have demonstrated infection with ABNC *V. cholerae* is possible without an *in vitro* pregrowth in rich media, but the doses used in these experiments were often quite high [14]. We do not propose diminishing the significance of the ABNC state as ABNC bacteria may still play a vital role in maintaining environmental reservoirs of facultative pathogens between outbreaks. However, our results indicate that the relevance of ABNC *V. cholerae* during an outbreak may be limited. These results are consistent with other systems that draw into question the role of ABNC cells in infection without *in vitro* pregrowth [61],[62].

In Dhaka, Bangladesh, lytic vibriophage are common in human patients and the environment [27],[28],[29]. The phage fluctuate in number seasonally in delayed concordance with cholera outbreaks [26],[27],[28], and household contacts of index cases that do not have lytic phage are at an increased risk of being infected with *V. cholerae* [29]. Despite this epidemiology, the dynamic role that phage play in the environment has not been studied. Phage carried over from the rice-water stool samples bloomed in the pond microcosm by 5 h. There was no significant rise in the phage titer between 5 and 24 h. However, the ratio of phage to CFU increased because of the continued decline in culturable counts between 5 and 24 h. Production of lytic phage is dependent on the growth of its host. Since the bacteria had no net increase/decrease in cell number in the pond system in the presence or absence of phage, it is likely that there was not sufficient growth capacity to make more phage. At the highest dose of *V. cholerae*, there was only partial colonization of mice infected with patient derived *V. cholerae* at 5 h, and fewer mice infected at 24 h. These data suggest that phage may reduce colonization, and at 24 h, the negative impact of phage on infectivity is exacerbated by the decline of culturable *V. cholerae*. Coinfection experiments with *V. cholerae* and lytic phage confirmed that phage have a negative impact on colonization. At low doses of bacteria and high doses of phage, the bacteria became more infectious. The relevance of this phenotype to the natural environment remains to be determined. The ready generation of LPS mutants in the coinfection experiment provides one mechanism by which bacteria may escape phage, but this is detrimental for the bacteria as LPS mutants are attenuated [63],[64]. This attenuation may be one reason why LPS mutants may not accumulate in the environment. Future studies to elucidate the mechanisms by which lytic phage may influence the infectivity of *V. cholerae* will add an additional dynamic to consider when modeling cholera transmission. At the most basic level, a better quantification of the infectious dose of *V. cholerae* in the natural setting, and the seasonal titer of phage and *V. cholerae* in the environment, will be a starting point for these future studies.

The microarray platform was similar to those previously used, but the method of analyzing the data by ANOVA and PCA to compare biological groups is novel for the *V. cholerae* field. ANOVA and PCA have been used in this manner in other fields when comparing large numbers of varied sample types [65],[66],[67]. qRT-PCR was used to validate the microarray. The results between qRT-PCR and the microarray were concordant; no false positives were observed by microarray when crosschecked with qRT-PCR. However as expected, the qRT-PCR was more sensitive than the microarray, and the opportunity for false negatives in the microarray analysis is therefore increased. The decrease in sensitivity by microarray was most pronounced with the patient derived samples after 24 h in pond water – with and without phage. One explanation for this decrease in sensitivity is that the RNA may be damaged at 24 h. If true, this would provide some insight into the physiological status of the bacteria at 24 h. qRT-PCR was normalized to an internal reference gene (*sanA* and *argS*) whereas the microarray was normalized to the global expression level. Therefore, qRT-PCR is less affected by RNA damage in this case because the references will theoretically also be equally affected by damaged RNA. If the RNA from cells incubated for 24 h is indeed damaged, this suggests that ABNC bacteria may be less capable of maintaining ribonucleic acid integrity. Despite these concerns, the congruence between the 5 h and 24 h samples at the global level demonstrates that the 24 h data are still informative albeit with reduced sensitivity at the level of individual genes.

The microarrays reveal immediate and striking transcriptional adjustment to the pond within the first 5 h. As the bacteria enter the ABNC state, they adapt to the nutrient limited nature of pond water by upregulating genes required for low phosphate and fixed nitrogen conditions. This adaptation is similar between patient and *in vitro* derived samples. In addition, there is a general down regulation of ribosomal proteins that indicates a general decline in the ability to synthesize new proteins. These congruent expression patterns are contrasted by divergent profiles of patient and *in vitro* derived samples at the global level. At both the 5 and 24 h time points, the patient derived samples do not converge with the *in vitro* derived samples by cluster analysis or by PCA. This result was not caused by the lytic phage in the patient derived samples because patient derived samples without phage were also divergent from the *in vitro* derived samples. These findings are supported by *in vitro* studies with lytic phage and *E. coli* and *P. aeruginosa* that detected changes in less than 4% of genes [68],[69].

The lack of convergence between patient and *in vitro* derived samples has relevance to vaccine development. One vaccine strategy is to vaccinate with *V. cholerae* in a ABNC state expressing ‘environmental’ surface proteins. The method behind this strategy was to transfer LB grown bacteria to pond water and allow the bacteria to enter the ABNC state and express a new repertoire of environmental antigens. This strategy is still valid, but we caution that *in vitro* derived cells incubated in pond water may indeed be ABNC, but they may express a different set of antigens than those from patients incubated in pond water. Thus, it may prove necessary to express surface proteins of ABNC bacteria derived from patients by genetic modification using inducible expression systems. Simple incubation of *in vitro* derived *V. cholerae* in pond water may not be adequate.

In summary, the dynamic interaction between lytic phage and bacteria in the pond environment suggests that the model of cholera transmission be reconsidered with respect to the urgency for transmission to the next host. Phage did not affect colonization immediately after shedding from the patients because the phage titer was too low. However, *V. cholerae* failed to colonize the animals after 24 h of incubation in pond water – the point when the phage and ABNC cell titers were highest. At 24 h, the rise of ABNC cells and lytic phage blocked transmission. The dialysis system was open with respect to small molecules but was closed with respect to the phage. The impact of the phage in the natural environment will be a function of the dilution of the bacteria away from the phage in large bodies of water that are free-flowing open systems such as rivers or closed systems such as ponds. Real-time studies of phage and bacterial titers in such bodies of water will provide a critical temporal factor to consider when gauging the negative selective pressure imposed by lytic phage and the ABNC state. Understanding this dynamic may ultimately demonstrate that the unfavorable conditions in the environment provide the critical selective pressure for toxigenic *V. cholerae* to maintain its facultative pathogen life-history strategy.

## Supporting Information

Figure S1 Hyperinfectivity is not induced *in vitro*.
(108 KB PDF)

Table S1 Bacterial strains and primers used in this study.
(13 KB PDF)

Table S2 Median qRT-PCR values per biological comparison (N = 3).
(68 KB PDF)

Table S3 Genes with differential expression (*P*<1×10^−7^) in at least one of the six conditions described in Fig. 6A–Node 1.
(20 KB PDF)

Table S4 Genes with differential expression (*P*<1×10^−7^) in at least one of the six conditions described in Fig. 6A–Node 2A.
(27 KB PDF)

Table S5 Genes with differential expression (*P*<1×10^−7^) in at least one of the six conditions described in Fig. 6A–Node 2B.
(22 KB PDF)

Table S6 Genes with differential expression (*P*<1×10^−7^) in at least one of the six conditions described in Fig. 6A–Node 3.
(32 KB PDF)

Table S7 Genes with differential expression (*P*<1×10^−7^) in at least one of the six conditions described in Fig. 6A–Node 4.
(18 KB PDF)

Table S8 Genes with differential expression (*P*<1×10^−7^) in at least one of the six conditions described in Fig. 7A–Node 1.
(32 KB PDF)

Table S9 Genes with differential expression (*P*<1×10^−7^) in at least one of the six conditions described in Fig. 7A–Node 2.
(16 KB PDF)

Table S10 Microarray data for the patient derived *V. cholerae* (phage+) vs. the *in vitro* derived *V. cholerae* comparison are provided in an Excel spreadsheet.
(50 KB PDF)
