# Supplementary material for: Transmission of Vibrio cholerae Is Antagonized by Lytic Phage and Entry into the Aquatic Environment
Source: PLoS Pathog. 2008 Oct 24;4(10):e1000187. doi: 10.1371/journal.ppat.1000187 (PMC2563029; doi:10.1371/journal.ppat.1000187)
Supplement: Figure S1 — Hyperinfectivity is not induced in vitro. (108 KB PDF) [file ppat.1000187.s001.doc]

**FIG. S1.**


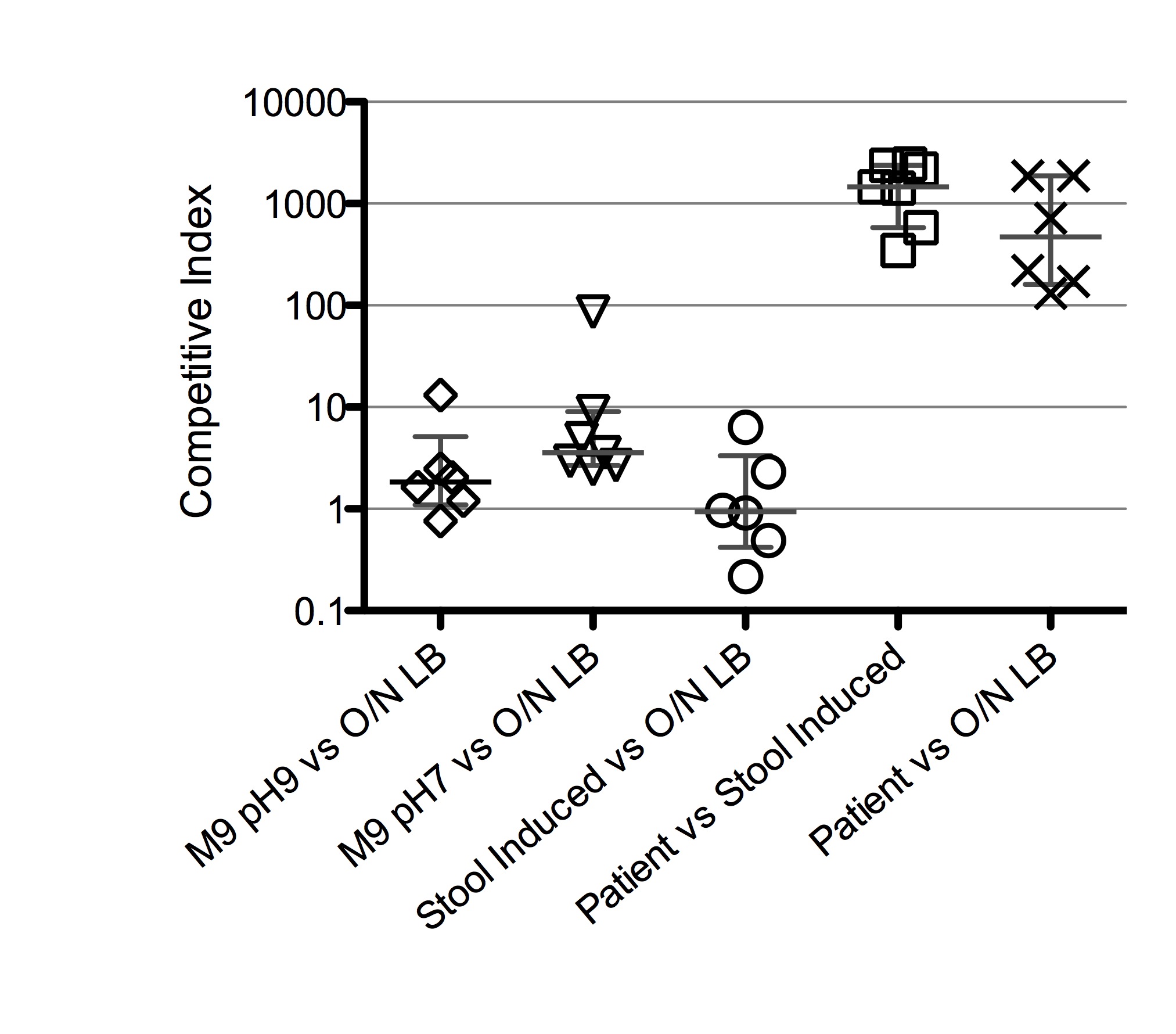


**Fig. S1. Hyperinfectivity is not induced *in vitro*.** *V. cholerae* (AC304; *lacZ*^+^) were incubated in M9 pH9 (diamonds) for 4 h, M9 pH7 (triangles) for 4 h, or 2 h in filter sterilized stool supernatant (circles) and competed against an overnight LB culture of El Tor *V. cholerae* (AC390; *lacZ*^-^) in the infant mouse model. Alternatively, patient derived rice-water stool *V. cholerae* was competed against *V. cholerae* incubated for 2 h in filter sterilized stool supernatant (squares) or overnight LB culture (crosses). Horizontal and vertical bars depict the median and inter-quartile range, respectively. The competitive index (CI) is calculated as the ratio of the mutant to the test strain after a 24 h infection -- corrected for the input ratio. Experiments with patient derived samples (squares and crosses) were not significantly different by the non-parametric Kruskal-Wallis test (α = 0.05) but were different from the other experimental groups (*P* ≤ 0.05). Competitions without patient derived samples were not significantly different between themselves (α = 0.05). Stool supernatant and patient derived *V. cholerae* were negative for lytic phage. These data represent two independent experiments.
