# Supplementary material for: Transmission of Vibrio cholerae Is Antagonized by Lytic Phage and Entry into the Aquatic Environment
Source: PLoS Pathog. 2008 Oct 24;4(10):e1000187. doi: 10.1371/journal.ppat.1000187 (PMC2563029; doi:10.1371/journal.ppat.1000187)
Supplement: Table S1 — Bacterial strains and primers used in this study. (13 KB PDF) [file ppat.1000187.s002.doc]

Supplementary Table S1. Bacterial strains and primers used in this study.

| Strain | Relevant Geneotype, or phenotype | Source/reference |
| --- | --- | --- |
| AC-304 | Wt N16961 (El Tor biotype, Inaba serotype) Sm^R^, lacZ^+^ | [1] |
| AC-390 | Derivative of AC-304, *lacZ-* | [2] |
| EN159^a^ | O1 El Tor biotype, Ogawa serotype, SM^R^, *lacZ+* | This work |
| EN182^a^ | O1 El Tor biotype, Inaba serotype, SM^S^, *lacZ+* | This work |
| EN191^a^, | O1 El Tor biotype, Inaba serotype, SM^R^, *lacZ+* | This work |
| EN124^b^ | O1 El Tor biotype, Ogawa serotype, SM^R^, *lacZ+* | This work |
| EN150^b^ | O1 El Tor biotype, Ogawa serotype, SM^R^, *lacZ+* | This work |
| EN174^b^ | O1 El Tor biotype, Ogawa serotype, SM^S^, *lacZ+* | This work |
|  |  |  |
| Primers | Sequence | Locus |
| *sanA* | Forward: 5’ TTGCTGTGGCTGACTATTGG 3’ | VC1186 |
|  | Reverse: 5’ CCAATACCACTGCAACCTGA 3’ |  |
| *argS* | Forward: 5’ TTCTCAGGCTATTGAAGCCG 3’ | VC2074 |
|  | Reverse: 5’ TAATGCCGTTGGCTTGGTAG 3’ |  |
| *cheW-1* | Forward: 5’ GGCATCAACGTAATGCAGGT | VC2059 |
|  | Reverse: 5’ AACAGTCACGACGTTACCAC |  |
| *cheY-4* | Forward: 5’ CATGCCAGTGATGACAGGTT | VCA1096 |
|  | Reverse: 5’ TTGACTAGCCATCCTGTTGC |  |
| *phoB* | Forward: 5’ AGCATATGAAGCGCGAAGAG 3’ | VC0719 |
|  | Reverse: 5’ ACGGGCAACCAACTCTTTAG 3’ |  |
| *glnB-1* | Forward: 5’ GGTGTCTGAAGTGAAAGGCT 3’ | VC0606 |
|  | Reverse: 5’ CCGCCTTAATGATCGCTTCA 3’ |  |
| *rplC* | Forward: 5’ TGAAACTGATGGCTACGCTG 3’ | VC2596 |
|  | Reverse: 5’ AACGGAATTCCCAAAGACCG 3’ |  |
| *rpoS* | Forward: 5’ ACCCAGATGTATCTCAGCGA 3’ | VC0534 |
|  | Reverse: 5’ CAATCATGCGTTTACGTGCG 3’ |  |

^a^ Strains isolated from rice-water stool samples that harbored phage at a ratio of at least 1000CFU ::1PFU. A paired lytic phage isolate was obtained from each stool sample.

^b^ Strains isolated from rice-water stool samples that did not harbor phage.

SUPPLEMENTAL REFERENCES

1. Heidelberg JF, Eisen JA, Nelson WC, Clayton RA, Gwinn ML, et al. (2000) DNA sequence of both chromosomes of the cholera pathogen *Vibrio cholerae*. Nature 406: 477-483.

2. Merrell DS, Hava DL, Camilli A (2002) Identification of novel factors involved in colonization and acid tolerance of *Vibrio cholerae*. Mol Microbiol 43: 1471-1491.
