# Supplementary material for: Transmission of Vibrio cholerae Is Antagonized by Lytic Phage and Entry into the Aquatic Environment
Source: PLoS Pathog. 2008 Oct 24;4(10):e1000187. doi: 10.1371/journal.ppat.1000187 (PMC2563029; doi:10.1371/journal.ppat.1000187)
Supplement: Table S2 — Median qRT-PCR values per biological comparison (N = 3). (68 KB PDF) [file ppat.1000187.s003.doc]

Supplementary Table S2. Median qRT-PCR values per biological comparison (N=3)^a^.

|  | *cheW-1* |  |  | *cheY-4* |  |  | *phoB* |  |  | *glnB-1* |  |  | *rplC* |  |  | *rpoS* |  |
| --- | --- | --- | --- | --- | --- | --- | --- | --- | --- | --- | --- | --- | --- | --- | --- | --- | --- |
|  | Phage^c^ | No Phage^c^ |  | Phage | No Phage |  | Phage | No Phage |  | Phage | No Phage |  | Phage | No Phage |  | Phage | No Phage |
| T0P/T0I^b^ | **-1.44** | **-2.27** |  | **-2.42** | **-2.35** |  | 0.41 | 0.81 |  | **-3.28** | **-2.31** |  | **1.40** | **2.03** |  | **-1.56** | **-1.25** |
| T5P/T5I | -0.89 |  |  | **-1.04** |  |  | **-1.07** |  |  | **1.52** |  |  | 0.01 |  |  | -0.13 |  |
| T24P/T24I | **-1.12** | -0.08 |  | **-1.41** | -0.79 |  | **-1.73** | **-1.77** |  | **1.19** | **1.19** |  | **-1.01** | -0.68 |  | **-1.23** | -0.73 |
|  |  |  |  |  |  |  |  |  |  |  |  |  |  |  |  |  |  |
| T5P/T0P | **2.06** | **1.01** |  | **3.01** | **1.56** |  | **2.49** | **4.33** |  | **5.68** | **5.91** |  | **-1.44** | **-1.80** |  | **2.68** | **1.84** |
| T24P/T0P | 0.93 | **2.88** |  | **3.01** | **5.27** |  | **1.81** | **4.64** |  | **2.19** | **7.26** |  | **-1.45** | **-1.96** |  | **1.83** | **2.35** |
| T24P/T5P | 0.04 | **3.19** |  | -0.06 | **4.24** |  | -0.66 | 0.03 |  | **-2.42** | **1.56** |  | -0.52 | **-1.15** |  | -0.68 | **1.29** |
|  |  |  |  |  |  |  |  |  |  |  |  |  |  |  |  |  |  |
| T5I/T0I | 0.93 |  |  | **1.61** |  |  | **5.29** |  |  | -0.38 |  |  | 0.81 |  |  | **1.12** |  |
| T24I/T0I | **0.04** | 0.69 |  | **1.28** | **1.23** |  | **4.93** | **5.17** |  | **-1.61** | **-2.62** |  | 0.14 | 0.78 |  | **1.19** | **1.24** |
| T24I/T5I | -0.62 |  |  | -0.43 |  |  | -0.28 |  |  | **-2.20** |  |  | -0.79 |  |  | 0.21 |  |
|  |  |  |  |  |  |  |  |  |  |  |  |  |  |  |  |  |  |
| T0P/LB ^d^ | **-2.98** |  |  | **4.41** |  |  | 0.62 |  |  | **3.40** |  |  | **-1.33** |  |  | **4.00** |  |

^a^ Values are the log_2_ of the median value (N=3). qRT-PCR was normalized against *argS* and *sanA*. Bold depicts a ≥ two-fold change (repression or induction).

^b^ Condition comparisons are as defined in Fig. 6 and Fig. 7. Sufficient sample was not available for T5I (no phage).

^c^ Defines if the original isolate came from a patient with phage (EN159, EN182, EN191) or no phage (EN124, EN150, EN174).

^d^ N16961 O1 El Tor *V. cholerae* was used as a reference comparison grown to mid-log (0.3 OD) in LB at 37ºC with aeration.
