# Supplementary material for: Transmission of Vibrio cholerae Is Antagonized by Lytic Phage and Entry into the Aquatic Environment
Source: PLoS Pathog. 2008 Oct 24;4(10):e1000187. doi: 10.1371/journal.ppat.1000187 (PMC2563029; doi:10.1371/journal.ppat.1000187)
Supplement: Table S3 — Genes with differential expression (P<1×10^−7^) in at least one of the six conditions described in Fig. 6A–Node 1. (20 KB PDF) [file ppat.1000187.s004.doc]

Supplementary Table S3. Genes with differential expression (*P* < 1 x 10^-7^) in at least one of the six conditions described in Fig. 6A – Node 1. Depicted is a rank of genes by major biological function followed by each individual gene grouped by function. In general, these genes were uniquely induced by patient derived *V. cholerae* in the aquatic environment. Table S10 provides gene specific fold-changes.

| Biological function | Number of genes | Percent of genes  with annotation | Genes of interest |
| --- | --- | --- | --- |
| Cellular processes | 5 | 15 |  |
| Energy Metabolism | 4 | 12 |  |
| Cell Envelope | 3 | 9 |  |
| Central metabolism | 3 | 9 |  |
| Biosynthesis of cofactors | 2 | 6 |  |
| DNA metabolism | 2 | 6 | *xerC, mutL* |
| Transport and binding proteins | 2 | 6 |  |
| Protein synthesis | 2 | 6 |  |
| Amino Acid Biosynthesis | 1 | 3 |  |
| Mobile and extrachromosomal role | 1 | 3 | *orfA* |
| Nucleic acid synthesis | 1 | 3 |  |
| Fatty acid metabolism | 0 | 0 |  |
| Protein fate | 0 | 0 |  |
| Regulation | 0 | 0 |  |
| Transcription | 0 | 0 |  |
|  |  |  |  |
| Hypothetical (annotated) | 8 | 24 |  |
| Total annotated genes | 34 | 100 |  |
| Hypotheticals (no annotation) | 6 |  |  |
|  |  |  |  |
| Cellular processes |  |  |  |
| Locus | Function | Gene | *P* Value |
| VC0282 | methyl-accepting chemotaxis protein |  | 5.1E-08 |
| VC0489 | hemolysin, putative |  | 2.1E-08 |
| VC1898 | methyl-accepting chemotaxis protein |  | 1.0E-13 |
| VCA0068 | methyl-accepting chemotaxis protein |  | 4.3E-09 |
| VCA0447 | haemagglutinin associated protein |  | 8.2E-08 |
|  |  |  |  |
| Energy Metabolism |  |  |  |
| Locus | Function | Gene | *P* Value |
| VC0793 | oxaloacetate decarboxylase, alpha subunit, authentic frameshift | *oadA-2* | 1.4E-08 |
| VC0797 | citrate lyase, gamma subunit | *citD* | 3.8E-09 |
| VC2293 | NADH:ubiquinone oxidoreductase, Na translocating, gamma subunit | *nqrC* | 3.1E-08 |
| VCA0155 | NADH dehydrogenase, putative |  | 2.4E-11 |

| Cell Envelope |  |  |  |
| --- | --- | --- | --- |
| Locus | Function | Gene | *P* Value |
| VC2407 | penicillin-binding protein 3 | *ftsI* | 1.3E-10 |
| VC2632 | fimbrial assembly protein PilO, putative |  | 3.7E-09 |
| VC2762 | UDP-N-acetylglucosamine pyrophosphorylase | *glmU* | 5.1E-08 |
|  |  |  |  |
| Central metabolism |  |  |  |
| Locus | Function | Gene | *P* Value |
| VC0994 | N-acetylglucosamine-6-phosphate deacetylase | *nagA-1* | 6.0E-08 |
| VC1591 | oxidoreductase, short-chain dehydrogenase-reductase family |  | 2.5E-08 |
| VC2559 | sulfate adenylate transferase, subunit 1 | *cysN* | 1.4E-08 |
|  |  |  |  |
| Biosynthesis of cofactors |  |  |  |
| Locus | Function | Gene | *P* Value |
| VC2718 | bioH protein | *bioH* | 6.5E-10 |
| VCA0827 | pterin-4-alpha-carbinolamine dehydratase | *phhB* | 6.5E-09 |
|  |  |  |  |
| DNA metabolism |  |  |  |
| Locus | Function | Gene | *P* Value |
| VC0128 | integrase-recombinase XerC | *xerC* | 5.3E-08 |
| VC0345 | DNA mismatch repair protein MutL | *mutL* | 2.4E-09 |
|  |  |  |  |
| Transport and binding proteins |  |  |  |
| Locus | Function | Gene | *P* Value |
| VC0433 | arginine-ornithine antiporter |  | 4.7E-08 |
| VCA0772 | tyrosine-specific transport protein | *tyrP* | 3.0E-10 |
|  |  |  |  |
| Protein synthesis |  |  |  |
| Locus | Function | Gene | *P* Value |
| VC0293 | ribosomal protein L11 methyltransferase | *prmA* | 1.4E-11 |
| VC2598 | RNA methyltransferase, TrmH family |  | 6.1E-10 |
|  |  |  |  |
| Amino Acid Biosynthesis |  |  |  |
| Locus | Function | Gene | *P* Value |
| VC2107 | aspartate-semialdehyde dehydrogenase, putative |  | 4.5E-09 |
|  |  |  |  |
| Mobile and extrachrom. Element fns |  |  |  |
| Locus | Function | Gene | *P* Value |
| VC0500 | transposase OrfAB, subunit A, authentic frameshift | *orfA* | 6.8E-08 |
|  |  |  |  |
| Nucleic acid synthesis |  |  |  |
| Locus | Function | Gene | *P* Value |
| VC2225 | uracil phosphoribosyltransferase | *upp* | 8.3E-12 |
|  |  |  |  |
| Hypothetical (annotated) |  |  |  |
| Locus | Function | Gene | *P* Value |
| VC0087 | conserved hypothetical protein |  | 3.1E-10 |
| VC1567 | conserved hypothetical protein |  | 1.4E-08 |
| VC1582 | conserved hypothetical protein |  | 2.6E-10 |
| VC2548 | conserved hypothetical protein |  | 1.2E-09 |
| VCA0355 | conserved hypothetical protein |  | 2.6E-09 |
| VCA0489 | conserved hypothetical protein |  | 5.2E-08 |
| VCA0847 | conserved hypothetical protein |  | 2.6E-08 |
| VCA1021 | conserved hypothetical protein |  | 8.2E-09 |
|  |  |  |  |
| Hypothetical (no annotation) |  |  |  |
| Locus | Function | Gene | *P* Value |
| VC0101 | hypothetical protein |  | 1.6E-08 |
| VC0383 | hypothetical protein |  | 2.1E-08 |
| VC0816 | hypothetical protein |  | 1.5E-09 |
| VC1387 | hypothetical protein |  | 3.2E-09 |
| VCA0399 | hypothetical protein |  | 6.4E-09 |
| VCA0599 | hypothetical protein |  | 2.5E-11 |
