# Supplementary material for: Transmission of Vibrio cholerae Is Antagonized by Lytic Phage and Entry into the Aquatic Environment
Source: PLoS Pathog. 2008 Oct 24;4(10):e1000187. doi: 10.1371/journal.ppat.1000187 (PMC2563029; doi:10.1371/journal.ppat.1000187)
Supplement: Table S4 — Genes with differential expression (P<1×10^−7^) in at least one of the six conditions described in Fig. 6A–Node 2A. (27 KB PDF) [file ppat.1000187.s005.doc]

Supplementary Table S4. Genes with differential expression (*P* < 1 x 10^-7^) in at least one of the six conditions described in Fig. 6A – Node 2A. Depicted is a rank of genes by major biological function followed by each individual gene grouped by function. In general, these genes were induced at all time points by *in vitro* derived *V. cholerae* in the aquatic environment. Table S10 provides gene specific fold-changes.

| Biological function | Number of genes | Percent of genes with annotation | Genes of interest |
| --- | --- | --- | --- |
| Cellular processes | 29 | 48 | Motiility , *rpoS, tcpP* |
| Regulation | 9 | 15 | *csrA, rseA* |
| Cell Envelope | 4 | 7 | *envA* |
| Amino Acid Biosynthesis | 1 | 2 |  |
| Central metabolism | 1 | 2 |  |
| Energy Metabolism | 1 | 2 |  |
| Mobile and extrachromosomal role | 1 | 2 | *rstR-2* |
| Transport and binding proteins | 1 | 2 |  |
| Protein fate | 1 | 2 |  |
| Transcription | 1 | 2 | *rpoH* |
| Biosyn of cofactors | 0 | 0 |  |
| DNA metabolism | 0 | 0 |  |
| Fatty acid metabolism | 0 | 0 |  |
| Protein synthesis | 0 | 0 |  |
| Nucleic acid synthesis | 0 | 0 |  |
|  |  |  |  |
| Hypothetical (annotated) | 11 | 18 |  |
| Total annotated genes | 60 | 100 |  |
| Hypotheticals (no annotation) | 28 |  |  |
|  |  |  |  |
| Cellular processes |  |  |  |
| Locus | Function | Gene | *P* Value |
| VC0534 | RNA polymerase sigma factor RpoS | *rpoS* | 2.5E-12 |
| VC0826 | toxin co-regulated pilus biosynthesis protein P | *tcpP* | 2.2E-15 |
| VC0892 | chemotaxis protein PomA | *pomA* | 2.9E-09 |
| VC1130 | DNA-binding protein VicH | *vicH* | 1.7E-16 |
| VC1456 | cholera enterotoxin, B subunit | *ctxB* | 1.9E-08 |
| VC2065 | chemotaxis protein CheY | *cheY-3* | 1.7E-15 |
| VC2069 | flagellar biosynthetic protein FlhA | *flhA* | 2.0E-08 |
| VC2128 | flagellar hook-length control protein FliK, putative |  | 1.6E-08 |
| VC2134 | flagellar hook-basal body complex protein FliE | *fliE* | 2.5E-15 |
| VC2138 | flagellar protein FliS | *fliS* | 6.8E-16 |
| VC2139 | flagellar rod protein FlaI, putative |  | 2.9E-09 |
| VC2142 | flagellin FlaB | *flaB* | 1.1E-08 |
| VC2143 | flagellin FlaD | *flaD* | 8.3E-14 |
| VC2144 | flagellin FlaE | *flaE* | 8.5E-12 |
| VC2187 | flagellin FlaC | *flaC* | 6.3E-12 |
| VC2188 | flagellin core protein A | *flaA* | 3.4E-10 |
| VC2191 | flagellar hook-associated protein FlgM | *flgM* | 3.9E-10 |
| VC2193 | flagellar P-ring protein FlgI | *flgI* | 2.5E-14 |
| VC2195 | flagellar basal-body rod protein FlgG | *flgG* | 4.2E-08 |
| VC2196 | flagellar basal-body rod protein FlgF | *flgF* | 1.2E-11 |
| VC2197 | flagellar hook protein FlgE | *flgE* | 5.4E-12 |
| VC2198 | basal-body rod modification protein FlgD | *flgD* | 8.1E-17 |
| VC2199 | flagellar basal-body rod protein FlgC | *flgC* | 1.3E-13 |
| VC2200 | flagellar basal-body rod protein FlgB | *flgB* | 1.1E-12 |
| VC2202 | chemotaxis protein CheV | *cheV-3* | 3.9E-08 |
| VC2204 | negative regulator of flagellin synthesis FlgM, putative |  | 1.1E-13 |
| VC2397 | cell division protein FtsZ | *ftsz* | 1.5E-11 |
| VC2398 | cell division protein FtsA | *ftsA* | 1.3E-10 |
| VC2601 | sodium-type flagellar protein MotX | *motX* | 1.0E-12 |
|  |  |  |  |
| Cellular processes |  |  |  |
| Locus | Function | Gene | *P* Value |
| VC0347 | host factor-I, putative |  | 6.7E-12 |
| VC0378 | zinc uptake regulation protein, putative |  | 1.8E-10 |
| VC0431 | arginine repressor | *argR* | 9.4E-08 |
| VC0548 | carbon storage regulator | *csrA* | 3.2E-11 |
| VC0583 | hemagglutinin-protease regulatory protein, authentic frameshift |  | 3.8E-14 |
| VC1434 | fumarate and nitrate reduction regulatory protein | *fnr* | 8.0E-08 |
| VC1914 | integration host factor, beta subunit | *hipB* | 7.5E-11 |
| VC2368 | aerobic respiration control protein FexA | *fexA* | 1.9E-09 |
| VC2466 | sigma-E factor negative regulatory protein RseA | *rseA* | 3.0E-14 |
|  |  |  |  |
| Cellular processes |  |  |  |
| Locus | Function | Gene | *P* Value |
| VC0845 | lipoprotein, putative |  | 7.9E-08 |
| VC1064 | lipoprotein-related protein |  | 4.0E-08 |
| VC1269 | lipoprotein, putative |  | 1.2E-13 |
| VC2396 | UDP-3-O-3-hydroxymyristoyl N-acetylglucosamine deacetylase | *envA* | 2.4E-11 |
|  |  |  |  |
| Amino Acid Biosynthesis |  |  |  |
| Locus | Function | Gene | *P* Value |
| VC2644 | N-acetyl-gamma-glutamyl-phosphate reductase | *argC* | 1.9E-08 |
|  |  |  |  |
| Central metabolism |  |  |  |
| Locus | Function | Gene | *P* Value |
| VC0252 | acetyltransferase RfbO, CysE-LacA-LpxA-NodL family |  | 6.2E-08 |
|  |  |  |  |
| Energy Metabolism |  |  |  |
| Locus | Function | Gene | *P* Value |
| VC0112 | cytochrome c4 | *cycA* | 4.3E-11 |
|  |  |  |  |
| Mobile and extrachrom. Element fns |  |  |  |
| Locus | Function | Gene | *P* Value |
| VC1464 | transcriptional repressor RstR | *rstR-2* | 3.4E-15 |
|  |  |  |  |
| Transport and binding proteins |  |  |  |
| Locus | Function | Gene | *P* Value |
| VC0964 | PTS system, glucose-specific IIA component | *crr* | 5.3E-09 |
|  |  |  |  |
| Protein fate |  |  |  |
| Locus | Function | Gene | *P* Value |
| VC0018 | 16 kDa heat shock protein A | *ibpA* | 3.6E-13 |
|  |  |  |  |
| Transcription |  |  |  |
| Locus | Function | Gene | *P* Value |
| VC0150 | RNA polymerase sigma-32 factor | *rpoH* | 3.7E-12 |
|  |  |  |  |
| Hypothetical (annotated) |  |  |  |
| Locus | Function | Gene | *P* Value |
| VC0049 | smg protein | *smg* | 3.3E-11 |
| VC2067 | MinD-related protein |  | 3.4E-09 |
| VCA0659 | protein F-related protein |  | 3.0E-08 |
| VC0079 | conserved hypothetical protein |  | 2.8E-12 |
| VC1124 | conserved hypothetical protein |  | 2.1E-15 |
| VC2040 | conserved hypothetical protein |  | 2.7E-13 |
| VC2478 | conserved hypothetical protein |  | 8.1E-12 |
| VCA0332 | conserved hypothetical protein |  | 7.6E-09 |
| VCA0741 | conserved hypothetical protein |  | 2.3E-09 |
| VCA0919 | conserved hypothetical protein |  | 1.3E-09 |
| VCA1042 | Ccm2-related protein |  | 1.2E-11 |
|  |  |  |  |
| Hypothetical (no annotation) |  |  |  |
| Locus | Function | Gene | *P* Value |
| VC0038 | hypothetical protein |  | 7.2E-13 |
| VC0174 | hypothetical protein |  | 5.5E-10 |
| VC0588 | hypothetical protein |  | 1.2E-08 |
| VC1125 | hypothetical protein |  | 4.1E-09 |
| VC1154 | hypothetical protein |  | 1.6E-08 |
| VC1384 | hypothetical protein |  | 1.8E-10 |
| VC1538 | hypothetical protein |  | 9.6E-12 |
| VC1613 | hypothetical protein |  | 3.2E-10 |
| VC1699 | hypothetical protein |  | 4.0E-10 |
| VC2005 | hypothetical protein |  | 4.5E-16 |
| VC2010 | hypothetical protein |  | 5.5E-16 |
| VC2189 | hypothetical protein |  | 7.1E-18 |
| VC2205 | hypothetical protein |  | 6.7E-14 |
| VC2207 | hypothetical protein |  | 7.4E-13 |
| VC2208 | hypothetical protein |  | 2.2E-08 |
| VC2263 | hypothetical protein |  | 2.5E-10 |
| VC2357 | hypothetical protein |  | 1.2E-08 |
| VC2365 | hypothetical protein |  | 3.4E-11 |
| VC2717 | hypothetical protein |  | 2.1E-12 |
| VCA0078 | hypothetical protein |  | 7.4E-11 |
| VCA0377 | hypothetical protein |  | 5.7E-08 |
| VCA0386 | hypothetical protein |  | 4.0E-08 |
| VCA0568 | hypothetical protein |  | 1.4E-10 |
| VCA0868 | hypothetical protein |  | 2.2E-17 |
| VCA0881 | hypothetical protein |  | 8.3E-08 |
| VCA0920 | hypothetical protein |  | 4.0E-08 |
| VCA1016 | hypothetical protein |  | 4.4E-17 |
| VCA1024 | hypothetical protein |  | 7.7E-14 |
