# Supplementary material for: Transmission of Vibrio cholerae Is Antagonized by Lytic Phage and Entry into the Aquatic Environment
Source: PLoS Pathog. 2008 Oct 24;4(10):e1000187. doi: 10.1371/journal.ppat.1000187 (PMC2563029; doi:10.1371/journal.ppat.1000187)
Supplement: Table S5 — Genes with differential expression (P<1×10^−7^) in at least one of the six conditions described in Fig. 6A–Node 2B. (22 KB PDF) [file ppat.1000187.s006.doc]

Supplementary Table S5. Genes with differential expression (*P* < 1 x 10^-7^) in at least one of the six conditions described in Fig. 6A – Node 2B. Depicted is a rank of genes by major biological function followed by each individual gene grouped by function. In general, these genes were uniquely induced by *in vitro* derived *V. cholerae* in the aquatic environment. Table S10 provides gene specific fold-changes.

| Biological function | Number of genes | Percent of genes with annotation | Genes of interest |
| --- | --- | --- | --- |
| Cellular processes | 14 | 29 | phage shock proteins, *cheY-4, cheR,* carbon starvation proteins |
| Energy Metabolism | 5 | 10 |  |
| Transport and binding proteins | 5 | 10 |  |
| Regulation | 5 | 10 |  |
| Amino Acid Biosynthesis | 3 | 6 |  |
| Mobile and extrachromosomal role | 2 | 4 | VCA0391 killer protein |
| Central metabolism | 1 | 2 |  |
| Fatty acid metabolism | 1 | 2 |  |
| Protein fate | 1 | 2 |  |
| Biosynthesis of cofactors | 0 | 0 |  |
| Cell Envelope | 0 | 0 |  |
| DNA metabolism | 0 | 0 |  |
| Protein synthesis | 0 | 0 |  |
| Nucleic acid synthesis | 0 | 0 |  |
| Transcription | 0 | 0 |  |
|  |  |  |  |
| Hypothetical (annotated) | 12 | 24 |  |
| Total annotated genes | 49 | 100 |  |
| Hypotheticals (no annotation) | 17 |  |  |
|  |  |  |  |
| Cellular processes |  |  |  |
| Locus | Function | Gene | *P* Value |
| VC0139 | DPS family protein |  | 3.0E-11 |
| VC0687 | carbon starvation protein A, putative |  | 2.2E-08 |
| VC1117 | heat shock protein HtpX | *htpX* | 3.2E-09 |
| VC1313 | methyl-accepting chemotaxis protein |  | 5.9E-11 |
| VC1399 | chemotaxis protein methyltransferase CheR | *cheR-1* | 6.7E-08 |
| VC1405 | methyl-accepting chemotaxis protein |  | 4.7E-14 |
| VC1677 | phage shock protein B | *pspB* | 2.2E-18 |
| VC1678 | phage shock protein A | *pspA* | 1.7E-23 |
| VCA0268 | methyl-accepting chemotaxis protein |  | 1.7E-10 |
| VCA0906 | methyl-accepting chemotaxis protein |  | 2.2E-08 |
| VCA0923 | methyl-accepting chemotaxis protein |  | 5.4E-11 |
| VCA1006 | organic hydroperoxide resistance protein, putative |  | 1.6E-10 |
| VCA1096 | chemotaxis protein CheY | *cheY-4* | 1.7E-15 |
| VCA1111 | thermostable hemolysin |  | 2.7E-08 |
|  |  |  |  |
| Energy Metabolism |  |  |  |
| Locus | Function | Gene | *P* Value |
| VC1336 | carboxyphosphonoenolpyruvate phosphonomutase | *prpB* | 5.0E-08 |
| VC1819 | aldehyde dehydrogenase | *aldA-2* | 6.7E-12 |
| VCA0644 | NADH oxidase, putative |  | 6.0E-13 |
| VCA0828 | phenylalanine-4-hydroxylase | *phhA* | 1.5E-13 |
| VCA1029 | glycogen operon protein GlgX | *glgX* | 3.4E-09 |
|  |  |  |  |
| Transport and binding proteins |  |  |  |
| Locus | Function | Gene | *P* Value |
| VC1549 | glycerol-3-phosphate ABC transporter, periplasmic glycerol-3-phosphate-binding protein | *ugpB* | 1.8E-09 |
| VC2215 | cation transport ATPase, E1-E2 family |  | 1.2E-08 |
| VC2705 | sodium-solute symporter, putative |  | 1.9E-09 |
| VCA0070 | phosphate ABC transporter, periplasmic phosphate-binding protein | *pstS* | 1.1E-18 |
| VCA0071 | phosphate ABC transporter, permease protein | *pstC-2* | 2.9E-10 |
|  |  |  |  |
| Regulation |  |  |  |
| Locus | Function | Gene | *P* Value |
| VC0719 | DNA-binding response regulator PhoB | *phoB* | 2.2E-14 |
| VC1335 | transcriptional regulator, GntR family |  | 4.8E-09 |
| VC1653 | sensory box sensor histidine kinase-response regulator VieS | *vieS* | 4.4E-08 |
| VC1741 | transcriptional regulator, TetR family |  | 1.7E-12 |
| VCA0642 | transcriptional regulator, ArsR family |  | 1.2E-14 |
|  |  |  |  |
| Amino Acid Biosynthesis |  |  |  |
| Locus | Function | Gene | *P* Value |
| VC1173 | anthranilate synthase component II | *trpG* | 3.6E-10 |
| VC2618 | acetylornithine aminotransferase | *argD* | 7.8E-09 |
| VC2642 | argininosuccinate synthase | *argG* | 9.7E-08 |
|  |  |  |  |
| Mobile and extrachrom. Element fns |  |  |  |
| Locus | Function | Gene | *P* Value |
| VCA0359 | plasmid stabilization element ParE, putative |  | 8.3E-10 |
| VCA0391 | killer protein, putative |  | 2.5E-10 |
|  |  |  |  |
| Central metabolism |  |  |  |
| Locus | Function | Gene | *P* Value |
| VC0748 | aminotransferase NifS, class V |  | 5.2E-08 |
|  |  |  |  |
| Fatty acid metabolism |  |  |  |
| Locus | Function | Gene | *P* Value |
| VC2758 | fatty oxidation complex, alpha subunit | *fadB* | 1.2E-08 |
|  |  |  |  |
| Protein fate |  |  |  |
| Locus | Function | Gene | *P* Value |
| VCA0639 | AcrA-AcrE family protein |  | 5.4E-08 |
|  |  |  |  |
| Hypothetical (annotated) |  |  |  |
| Locus | Function | Gene | *P* Value |
| VC0749 | NifU-related protein |  | 5.3E-12 |
| VC0750 | hesB family protein |  | 4.1E-10 |
| VC0747 | conserved hypothetical protein |  | 1.2E-13 |
| VC1099 | conserved hypothetical protein |  | 1.8E-10 |
| VC1872 | conserved hypothetical protein |  | 5.5E-11 |
| VC2340 | conserved hypothetical protein |  | 2.0E-09 |
| VC2473 | conserved hypothetical protein |  | 6.4E-15 |
| VC2488 | conserved hypothetical protein |  | 3.4E-17 |
| VC2507 | conserved hypothetical protein |  | 7.2E-12 |
| VCA0641 | conserved hypothetical protein |  | 1.5E-14 |
| VCA0716 | conserved hypothetical protein |  | 1.1E-10 |
| VCA0948 | conserved hypothetical protein |  | 2.7E-14 |
|  |  |  |  |
| Hypothetical (no annotation) |  |  |  |
| Locus | Function | Gene | *P* Value |
| VC0025 | hypothetical protein |  | 8.6E-18 |
| VC0978 | hypothetical protein |  | 1.8E-12 |
| VC1116 | hypothetical protein |  | 9.1E-18 |
| VC1323 | hypothetical protein |  | 1.7E-11 |
| VC1368 | hypothetical protein |  | 1.1E-22 |
| VC1661 | hypothetical protein |  | 7.5E-12 |
| VC1932 | hypothetical protein |  | 3.8E-12 |
| VC1933 | hypothetical protein |  | 1.4E-09 |
| VC2667 | hypothetical protein |  | 4.7E-09 |
| VC2704 | hypothetical protein |  | 7.5E-09 |
| VCA0004 | hypothetical protein |  | 3.3E-10 |
| VCA0087 | hypothetical protein |  | 1.1E-12 |
| VCA0333 | hypothetical protein |  | 1.3E-08 |
| VCA0360 | hypothetical protein |  | 6.4E-10 |
| VCA0551 | hypothetical protein |  | 2.5E-16 |
| VCA0715 | hypothetical protein |  | 8.4E-13 |
| VCA0994 | hypothetical protein |  | 1.5E-17 |
