# Supplementary material for: Transmission of Vibrio cholerae Is Antagonized by Lytic Phage and Entry into the Aquatic Environment
Source: PLoS Pathog. 2008 Oct 24;4(10):e1000187. doi: 10.1371/journal.ppat.1000187 (PMC2563029; doi:10.1371/journal.ppat.1000187)
Supplement: Table S7 — Genes with differential expression (P<1×10^−7^) in at least one of the six conditions described in Fig. 6A–Node 4. (18 KB PDF) [file ppat.1000187.s008.doc]

Supplementary Table S7. Genes with differential expression (*P* < 1 x 10^-7^) in at least one of the six conditions described in Fig. 6A – Node 4. Depicted is a rank of genes by major biological function followed by each individual gene grouped by function. In general, these genes were induced by both patient and *in vitro* derived *V. cholerae* at 0 h. A smaller clade of genes were only induced in the *in vitro* derived *V. cholerae* at 0 h; these genes were largely involved in glycerol metabolism because the minimal media contained glycerol. By 24 h, all genes in this node were repressed. Table S10 provides gene specific fold-changes.

| Biological function | Number of genes | Percent of genes with annotation | Genes of interest |
| --- | --- | --- | --- |
| Energy Metabolism | 13 | 35 |  |
| Transport and binding proteins | 6 | 16 |  |
| Regulation | 3 | 8 | sigma54 |
| Biosynthesis of cofactors | 2 | 5 |  |
| Cellular processes | 2 | 5 |  |
| Cell Envelope | 1 | 3 | *ompU* |
| Central metabolism | 1 | 3 |  |
| Protein synthesis | 1 | 3 |  |
| Nucleic acid synthesis | 1 | 3 |  |
| Amino Acid Biosynthesis | 0 | 0 |  |
| DNA metabolism | 0 | 0 |  |
| Mobile and extrachromosomal role | 0 | 0 |  |
| Fatty acid metabolism | 0 | 0 |  |
| Protein fate | 0 | 0 |  |
| Transcription | 0 | 0 |  |
|  |  |  |  |
| Hypothetical (annotated) | 7 | 19 |  |
| Total annotated genes | 37 | 100 |  |
| Hypothetical (no annotation) | 12 |  |  |
|  |  |  |  |
| Energy Metabolism |  |  |  |
| Locus | Function | Gene | *P* Value |
| VC0089 | cytochrome c551 peroxidase | *yhjA* | 1.8E-08 |
| VC1866 | formate acetyltransferase | *pflB* | 2.5E-08 |
| VC2241 | cytochrome c554 |  | 7.7E-08 |
| VC2656 | fumarate reductase, flavoprotein subunit | *frdA* | 8.8E-18 |
| VC2657 | fumarate reductase, iron-sulfur protein | *frdB* | 3.9E-23 |
| VC2658 | fumarate reductase, 15 kDa hydrophobic protein | *frdC* | 2.2E-17 |
| VC2659 | fumarate reductase, 13 kDa hydrophobic protein | *frdD* | 6.6E-16 |
| VC2738 | phosphoenolpyruvate carboxykinase | *pckA* | 2.6E-14 |
| VCA0702 | iron-containing alcohol dehydrogenase |  | 2.1E-08 |
| VCA0744 | glycerol kinase | *gplK* | 1.7E-10 |
| VCA0747 | anaerobic glycerol-3-phosphate dehydrogenase, subunit A | *glpA* | 3.5E-14 |
| VCA0748 | anaerobic glycerol-3-phosphate dehydrogenase, subunit B | *glpB* | 3.9E-11 |
|  |  |  |  |
| Transport and binding proteins |  |  |  |
| Locus | Function | Gene | *P* Value |
| VC0010 | amino acid ABC transporter, periplasmic amino acid-binding portion |  | 2.9E-09 |
| VC0069 | multidrug resistance protein, putative |  | 1.1E-08 |
| VC1929 | C4-dicarboxylate-binding periplasmic protein | *dctP-2* | 3.4E-10 |
| VCA0137 | glycerol-3-phosphate transporter | *glpT* | 1.4E-14 |
| VCA0205 | C4-dicarboxylate transporter, anaerobic | *dcuB* | 4.0E-10 |
| VCA0745 | glycerol uptake facilitator protein, authentic frameshift | *glpF* | 2.1E-10 |
|  |  |  |  |
| Regulation |  |  |  |
| Locus | Function | Gene | *P* Value |
| VC0330 | regulator of sigma D | *rsd* | 1.6E-08 |
| VC0706 | sigma-54 modulation protein, putative |  | 4.3E-16 |
| VC1082 | response regulator |  | 1.2E-09 |
|  |  |  |  |
| Biosynthesis of cofactors |  |  |  |
| Locus | Function | Gene | *P* Value |
| VC1973 | naphthoate synthase | *menB* | 5.8E-09 |
| VCA0610 | sigma cross-reacting protein 27A |  | 3.0E-09 |
|  |  |  |  |
| Cellular processes |  |  |  |
| Locus | Function | Gene | *P* Value |
| VC1316 | chemotaxis protein CheY, putative |  | 7.4E-09 |
| VCA0594 | hemolysin | *hlx* | 4.7E-10 |
|  |  |  |  |
| Cell Envelope |  |  |  |
| Locus | Function | Gene | *P* Value |
| VC0633 | outer membrane protein OmpU | *ompU* | 2.1E-21 |
|  |  |  |  |
| Central metabolism |  |  |  |
| Locus | Function | Gene | *P* Value |
| VCA0798 | CbbY family protein |  | 7.0E-10 |
|  |  |  |  |
| Protein synthesis |  |  |  |
| Locus | Function | Gene | *P* Value |
| VC1484 | ribosome modulation factor | *rmf* | 1.1E-19 |
|  |  |  |  |
| Nucleic acid synthesis |  |  |  |
| Locus | Function | Gene | *P* Value |
| VC0052 | phosphoribosylaminoimidazole carboxylase, catalytic subunit | *purE* | 4.7E-14 |
|  |  |  |  |
| Hypothetical (annotated) |  |  |  |
| Locus | Function | Gene | *P* Value |
| VC2361 | formate acetyl transferase-related protein |  | 2.8E-18 |
| VC0146 | conserved hypothetical protein |  | 5.7E-10 |
| VC0957 | conserved hypothetical protein |  | 1.9E-13 |
| VC1153 | conserved hypothetical protein |  | 8.0E-12 |
| VC1249 | conserved hypothetical protein |  | 9.2E-12 |
| VC1871 | conserved hypothetical protein |  | 5.4E-08 |
| VCA1065 | conserved hypothetical protein |  | 1.0E-09 |
|  |  |  |  |
| Hypothetical (no annotation) |  |  |  |
| Locus | Function | Gene | *P* Value |
| VC0016 | hypothetical protein |  | 1.3E-15 |
| VC0017 | hypothetical protein |  | 6.0E-16 |
| VC0413 | hypothetical protein |  | 1.5E-08 |
| VC1189 | hypothetical protein |  | 4.0E-14 |
| VC1224 | hypothetical protein |  | 1.6E-08 |
| VC2221 | hypothetical protein |  | 2.8E-19 |
| VC2543 | hypothetical protein |  | 1.0E-09 |
| VC2609 | hypothetical protein |  | 3.0E-08 |
| VCA0882 | hypothetical protein |  | 1.1E-12 |
| VCA0883 | hypothetical protein |  | 1.2E-08 |
| VCA0884 | hypothetical protein |  | 2.3E-11 |
| VCA0892 | hypothetical protein |  | 1.8E-08 |
