# Supplementary material for: Transmission of Vibrio cholerae Is Antagonized by Lytic Phage and Entry into the Aquatic Environment
Source: PLoS Pathog. 2008 Oct 24;4(10):e1000187. doi: 10.1371/journal.ppat.1000187 (PMC2563029; doi:10.1371/journal.ppat.1000187)
Supplement: Table S8 — Genes with differential expression (P<1×10^−7^) in at least one of the six conditions described in Fig. 7A–Node 1. (32 KB PDF) [file ppat.1000187.s009.doc]

Supplementary Table S8. Genes with differential expression (*P* < 1 x 10^-7^) in at least one of the six conditions described in Fig. 7A – Node 1. Depicted is a rank of genes by major biological function followed by each individual gene grouped by function. In general, these genes were induced at 0 h and were repressed by 24 h in the aquatic environment.

| Biological function | Number of genes | Percent of genes with annotation | Genes of interest |
| --- | --- | --- | --- |
| Protein synthesis | 60 | 42 |  |
| Energy Metabolism | 17 | 12 |  |
| Cellular process | 12 | 8 | flagellar genes, *fts, ssp, cheZ* |
| Cell Envelope | 10 | 7 | *ompU, envA, ompW, ompA, ompH* |
| Regulation | 8 | 6 |  |
| Transcription | 7 | 5 | *rpoA, rpoH, rpoH* |
| Fatty acid metabolism | 5 | 3 |  |
| Protein fate | 5 | 3 | sec and tat secretion systems |
| Transport and binding proteins | 3 | 2 | *ompT* |
| Biosynthesis of cofactors | 2 | 1 |  |
| Central metabolism | 2 | 1 |  |
| DNA metabolism | 2 | 1 |  |
| Nucleic acid synthesis | 2 | 1 |  |
|  |  |  |  |
| Hypothetical (annotated) | 8 | 6 |  |
| Total annotated genes | 143 |  |  |
| Hypothetical (no annotation) | 9 |  |  |
|  |  |  |  |
| Protein synthesis |  |  |  |
| Locus | Gene | Function | *P* Value |
| VC0218 | ribosomal protein L28 | *rpmB* | 6.1E-10 |
| VC0219 | ribosomal protein L33 | *rpmG* | 1.7E-09 |
| VC0321 | elongation factor Tu | *tufB* | 2.7E-12 |
| VC0324 | ribosomal protein L11 | *rplK* | 8.9E-10 |
| VC0325 | ribosomal protein L1 | *rplA* | 3.9E-13 |
| VC0326 | ribosomal protein L10 | *rplJ* | 5.4E-19 |
| VC0327 | ribosomal protein L7-L12 | *rplL* | 1.6E-13 |
| VC0359 | ribosomal protein S12 | *rpsL* | 4.4E-11 |
| VC0360 | ribosomal protein S7 | *rpsG* | 6.8E-10 |
| VC0361 | elongation factor G | *fusA-1* | 2.0E-09 |
| VC0366 | ribosomal protein S6 | *rpsF* | 2.3E-17 |
| VC0368 | ribosomal protein S18 | *rpsR* | 2.5E-15 |
| VC0369 | ribosomal protein L9 | *rplI* | 1.5E-12 |
| VC0436 | ribosomal protein L27 | *rpmA* | 3.4E-11 |
| VC0520 | ribosomal protein S21 | *rpsU* | 1.5E-08 |
| VC0561 | ribosomal protein S16 | *rpsP* | 7.1E-09 |
| VC0563 | tRNA (guanine-N1)-methyltransferase | *trmD* | 1.5E-11 |
| VC0564 | ribosomal protein L19 | *rplS* | 6.1E-13 |
| VC0570 | ribosomal protein L13 | *rplM* | 5.2E-12 |
| VC0571 | ribosomal protein S9 | *rpsI* | 8.5E-15 |
| VC0643 | initiation factor IF-2 | *infB* | 3.1E-09 |
| VC0645 | tRNA pseudouridine 55 synthase | *truB* | 1.0E-08 |
| VC0646 | ribosomal protein S15 | *rpsO* | 1.3E-10 |
| VC0682 | isoleucyl-tRNA synthetase | *ileS* | 4.5E-08 |
| VC1179 | pseudouridine synthase family 1 protein |  | 1.8E-08 |
| VC1640 | ribosomal protein L25 | *rplY* | 3.5E-13 |
| VC1915 | ribosomal protein S1 | *rpsA* | 4.4E-12 |
| VC2025 | ribosomal protein L32 | *rpmF* | 1.8E-10 |
| VC2257 | ribosome recycling factor | *frr* | 2.2E-09 |
| VC2260 | ribosomal protein S2 | *rpsB* | 1.3E-13 |
| VC2570 | ribosomal protein L17 | *rplQ* | 2.3E-09 |
| VC2572 | ribosomal protein S4 | *rpsD* | 7.0E-11 |
| VC2573 | ribosomal protein S11 | *rpsK* | 2.4E-16 |
| VC2574 | ribosomal protein S13 | *rpsM* | 5.7E-16 |
| VC2575 | ribosomal protein L36 | *rpmJ* | 3.6E-16 |
| VC2577 | ribosomal protein L15 | *rplO* | 1.7E-19 |
| VC2578 | ribosomal protein L30 | *rpmD* | 8.6E-17 |
| VC2579 | ribosomal protein S5 | *rpsE* | 2.0E-09 |
| VC2580 | ribosomal protein L18 | *rplR* | 1.3E-19 |
| VC2581 | ribosomal protein L6 | *rplF* | 4.5E-14 |
| VC2582 | ribosomal protein S8 | *rpsH* | 3.1E-11 |
| VC2583 | ribosomal protein S14 | *rpsN* | 4.1E-20 |
| VC2584 | ribosomal protein L5 | *rplE* | 4.1E-14 |
| VC2585 | ribosomal protein L24 | *rplX* | 1.3E-18 |
| VC2586 | ribosomal protein L14 | *rplN* | 1.8E-13 |
| VC2587 | ribosomal protein S17 | *rpsQ* | 1.4E-17 |
| VC2588 | ribosomal protein L29 | *rpmC* | 4.9E-20 |
| VC2589 | ribosomal protein L16 | *rplP* | 1.1E-17 |
| VC2590 | ribosomal protein S3 | *rpsC* | 3.2E-14 |
| VC2591 | ribosomal protein L22 | *rplV* | 1.1E-23 |
| VC2592 | ribosomal protein S19 | *rpsS* | 4.1E-13 |
| VC2593 | ribosomal protein L2 | *rplB* | 1.5E-17 |
| VC2594 | ribosomal protein L23 | *rplW* | 3.9E-16 |
| VC2595 | ribosomal protein L4 | *rplD* | 1.9E-13 |
| VC2596 | ribosomal protein L3 | *rplC* | 3.5E-15 |
| VC2597 | ribosomal protein S10 | *rpsJ* | 3.4E-11 |
| VC2660 | elongation factor P | *efp* | 1.5E-12 |
| VC2679 | ribosomal protein L31 | *rpmE* | 5.3E-12 |
| VCA0289 | ribosomal protein L35 | *rpmI* | 6.6E-09 |
| VCA0290 | ribosomal protein L20 | *rplT* | 1.1E-14 |
|  |  |  |  |
| Energy Metabolism |  |  |  |
| Locus | Gene | Function | *P* Value |
| VC0112 | cytochrome c4 | *cycA* | 1.0E-09 |
| VC0575 | ubiquinol--cytochrome c reductase, cytochrome c1 | *petC* | 4.8E-12 |
| VC1146 | glutaredoxin 1 | *grxA* | 2.7E-11 |
| VC1441 | cytochrome c oxidase, subunit CcoO | *ccoO* | 6.2E-11 |
| VC2092 | citrate synthase | *gltA* | 7.7E-13 |
| VC2290 | NADH:ubiquinone oxidoreductase, Na translocating, beta subunit | *nqrF* | 5.1E-15 |
| VC2291 | NADH:ubiquinone oxidoreductase, Na translocating, hydrophobic membrane protein NqrE | *nqrE* | 1.5E-08 |
| VC2295 | NADH:ubiquinone oxidoreductase, Na translocating, alpha subunit | *nqrA* | 3.8E-08 |
| VC2480 | ribose-5-phosphate isomerase | *rpiA* | 6.1E-08 |
| VC2659 | fumarate reductase, 13 kDa hydrophobic protein | *frdD* | 7.7E-08 |
| VC2738 | phosphoenolpyruvate carboxykinase | *pckA* | 9.5E-09 |
| VC2764 | ATP synthase F1, beta subunit | *atpD* | 5.4E-12 |
| VC2765 | ATP synthase F1, gamma subunit | *atpG* | 2.7E-11 |
| VC2768 | ATP synthase F0, B subunit | *atpF* | 3.4E-09 |
| VC2769 | ATP synthase F0, C subunit | *atpE* | 7.3E-08 |
| VC2770 | ATP synthase F0, A subunit | *atpB* | 4.7E-09 |
| VCA0843 | glyceraldehyde 3-phosphate dehydrogenase | *gapA-2* | 8.9E-08 |
|  |  |  |  |
| Cellular processes |  |  |  |
| Locus | Gene | Function | *P* Value |
| VC0139 | DPS family protein |  | 7.0E-08 |
| VC0576 | stringent starvation protein A | *sspA* | 2.3E-10 |
| VC0577 | stringent starvation protein B | *sspB* | 5.6E-11 |
| VC1289 | methyl-accepting chemotaxis protein |  | 3.0E-08 |
| VC2064 | chemotaxis protein CheZ | *cheZ* | 6.5E-11 |
| VC2143 | flagellin FlaD | *flaD* | 1.3E-09 |
| VC2187 | flagellin FlaC | *flaC* | 3.4E-09 |
| VC2193 | flagellar P-ring protein FlgI | *flgI* | 8.2E-09 |
| VC2198 | basal-body rod modification protein FlgD | *flgD* | 8.7E-09 |
| VC2199 | flagellar basal-body rod protein FlgC | *flgC* | 1.5E-08 |
| VC2397 | cell division protein FtsZ | *ftsz* | 8.5E-08 |
| VC2398 | cell division protein FtsA | *ftsA* | 7.1E-12 |
|  |  |  |  |
| Cell Envelope |  |  |  |
| Locus | Gene | Function | *P* Value |
| VC0243 | GDP-mannose 4,6-dehydratase | *rfbD* | 3.1E-08 |
| VC0633 | outer membrane protein OmpU | *ompU* | 9.5E-13 |
| VC1835 | peptidoglycan-associated lipoprotein | *pal* | 1.8E-08 |
| VC2156 | lipoprotein-34 NlpB | *nlpB* | 7.2E-08 |
| VC2175 | 2-dehydro-3-deoxyphosphooctonate aldolase | *kdsA* | 4.2E-11 |
| VC2213 | outer membrane protein OmpA | *ompA* | 2.2E-08 |
| VC2250 | UDP-3-O-3-hydroxymyristoyl glucosamine N-acyltransferase | *lpxD* | 4.2E-12 |
| VC2251 | outer membrane protein OmpH | *ompH* | 2.1E-08 |
| VC2396 | UDP-3-O-3-hydroxymyristoyl N-acetylglucosamine deacetylase | *envA* | 2.6E-09 |
| VCA0867 | outer membrane protein OmpW | *ompW* | 2.2E-12 |
|  |  |  |  |
| Regulation | |  |  |
| Locus | Gene | Function | *P* Value |
| VC0290 | factor-for-inversion stimulation protein | *fis* | 1.2E-11 |
| VC0378 | zinc uptake regulation protein, putative |  | 4.4E-09 |
| VC0583 | hemagglutinin-protease regulatory protein, authentic frameshift |  | 6.2E-10 |
| VC2106 | ferric uptake regulation protein | *fur* | 1.5E-10 |
| VC2368 | aerobic respiration control protein FexA | *fexA* | 1.9E-08 |
| VCA0166 | cold shock transcriptional regulator CspA | *cspA* | 5.5E-09 |
| VCA0184 | cold shock DNA-binding domain protein |  | 3.5E-10 |
| VCA0933 | cold shock domain family protein |  | 5.2E-11 |
|  |  |  |  |
| Transcription |  |  |  |
| Locus | Gene | Function | *P* Value |
| VC0150 | RNA polymerase sigma-32 factor | *rpoH* | 3.6E-09 |
| VC0307 | transcription termination factor Rho | *rho* | 1.5E-12 |
| VC0323 | transcription antitermination protein NusG | *nusG* | 3.2E-09 |
| VC0328 | DNA-directed RNA polymerase, beta subunit | *rpoB* | 9.0E-08 |
| VC0562 | 16S rRNA processing protein RimM | *rimM* | 1.3E-13 |
| VC2571 | DNA-directed RNA polymerase, alpha subunit | *rpoA* | 1.0E-17 |
| VCA0804 | ATP-dependent RNA helicase DeaD | *deaD* | 4.7E-12 |
|  |  |  |  |
| Fatty acid metabolism |  |  |  |
| Locus | Gene | Function | *P* Value |
| VC0251 | acyl protein synthase-acyl-CoA reductase RfbN |  | 8.1E-08 |
| VC0745 | inositol monophosphate family protein |  | 3.0E-09 |
| VC2021 | 3-oxoacyl-(acyl-carrier-protein) reductase | *fabG* | 2.3E-10 |
| VC2022 | malonyl CoA-acyl carrier protein transacylase | *fabD* | 1.8E-08 |
| VC2249 | (3R)-hydroxymyristoyl-(acyl-carrier-protein) dehydratase | *fabZ* | 2.0E-09 |
|  |  |  |  |
| Protein fate |  |  |  |
| Locus | Gene | Function | *P* Value |
| VC0086 | tatA protein | *tatA-1* | 5.8E-10 |
| VC0322 | preprotein translocase, SecE subunit | *secE* | 1.3E-09 |
| VC0640 | preprotein translocase, SecG subunit | *secG* | 4.3E-09 |
| VC0744 | protein-export membrane protein SecF | *secF-1* | 8.8E-08 |
| VC2576 | preprotein translocase, SecY subunit | *secY* | 1.6E-17 |
|  |  |  |  |
| Transport and binding proteins |  |  |  |
| Locus | Gene | Function | *P* Value |
| VC1042 | long-chain fatty acid transport protein | *fadL-1* | 5.2E-09 |
| VC1091 | oligopeptide ABC transporter, periplasmic oligopeptide-binding protein | *oppA* | 3.9E-10 |
| VC1854 | ompT protein | *ompT* | 4.7E-08 |
|  |  |  |  |
| Biosynthesis of cofactors |  |  |  |
| Locus | Gene | Function | *P* Value |
| VC0440 | dihydrofolate reductase | *folA* | 3.5E-08 |
| VC2268 | 6,7-dimethyl-8-ribityllumazine synthase | *ribE* | 2.5E-08 |
|  |  |  |  |
| Central metabolism |  |  |  |
| Locus | Gene | Function | *P* Value |
| VC2545 | inorganic pyrophosphatase | *ppa* | 3.2E-08 |
| VCA0341 | biphenyl-2,3-diol 1,2-dioxygenase III-related protein |  | 1.7E-08 |
|  |  |  |  |
| DNA metabolism |  |  |  |
| Locus | Gene | Function | *P* Value |
| VC2545 | inorganic pyrophosphatase | *ppa* | 3.2E-08 |
| VCA0341 | biphenyl-2,3-diol 1,2-dioxygenase III-related protein |  | 1.7E-08 |
|  |  |  |  |
| Nucleic acid synthesis |  |  |  |
| Locus | Gene | Function | *P* Value |
| VC0052 | phosphoribosylaminoimidazole carboxylase, catalytic subunit | *purE* | 6.6E-09 |
| VC0395 | UTP--glucose-1-phosphate uridylyltransferase | *galU* | 7.0E-08 |
|  |  |  |  |
| Hypothetical (annotated) |  |  |  |
| Locus | Gene | Function | *P* Value |
| VC0253 | IS1004 transposase-related protein |  | 3.6E-08 |
| VC2067 | MinD-related protein |  | 3.1E-08 |
| VC2688 | glpX protein | *glpX* | 3.1E-09 |
| VC0641 | conserved hypothetical protein |  | 1.2E-11 |
| VC2026 | conserved hypothetical protein |  | 7.1E-13 |
| VC2326 | conserved hypothetical protein |  | 4.9E-10 |
| VC2443 | conserved hypothetical protein |  | 2.0E-09 |
| VCA0037 | conserved hypothetical protein |  | 4.9E-09 |
|  |  |  |  |
| Hypothetical (no annotation) |  |  |  |
| Locus | Gene | Function | *P* Value |
| VC0059 | hypothetical protein |  | 1.0E-10 |
| VC0895 | hypothetical protein |  | 1.5E-08 |
| VC1517 | hypothetical protein |  | 4.3E-08 |
| VC1752 | hypothetical protein |  | 5.7E-09 |
| VC2208 | hypothetical protein |  | 3.0E-08 |
| VC2496 | hypothetical protein |  | 9.8E-10 |
| VCA0306 | hypothetical protein |  | 4.5E-08 |
| VCA0464 | hypothetical protein |  | 7.3E-10 |
| VCA0502 | hypothetical protein |  | 3.1E-09 |
