# Supplementary material for: Transmission of Vibrio cholerae Is Antagonized by Lytic Phage and Entry into the Aquatic Environment
Source: PLoS Pathog. 2008 Oct 24;4(10):e1000187. doi: 10.1371/journal.ppat.1000187 (PMC2563029; doi:10.1371/journal.ppat.1000187)
Supplement: Table S9 — Genes with differential expression (P<1×10^−7^) in at least one of the six conditions described in Fig. 7A–Node 2. (16 KB PDF) [file ppat.1000187.s010.doc]

Supplementary Table S9. Genes with differential expression (*P* < 1 x 10^-7^) in at least one of the six conditions described in Fig. 7A – Node 2. Depicted is a rank of genes by major biological function followed by each individual gene grouped by function. In general, these genes were repressed at 0 h and were induced by 24 h in the aquatic environment.

| Biological function | Number of genes | Percent of genes with annotation | Genes of interest |
| --- | --- | --- | --- |
| Transport and binding proteins | 5 | 23 |  |
| Cellular processes | 4 | 18 | *cheY-4* |
| Cell Envelope | 2 | 9 | fimbrial proteins |
| Biosynthesis of cofactors | 1 | 5 |  |
| Central metabolism | 1 | 5 |  |
| DNA metabolism | 1 | 5 |  |
| Energy Metabolism | 1 | 5 |  |
| Fatty acid metabolism | 0 | 0 |  |
| Protein fate | 0 | 0 |  |
| Protein synthesis | 0 | 0 |  |
| Nucleic acid synthesis | 0 | 0 |  |
| Regulation | 0 | 0 |  |
| Transcription | 0 | 0 |  |
|  |  |  |  |
| Hypothetical (annotated) | 7 | 32 |  |
| Total annotated genes | 22 |  |  |
| Hypotheticals (no annotation) | 8 |  |  |
|  |  |  |  |
| Transport and binding proteins |  |  |  |
| Locus | Function | Gene | *P* Value |
| VC1547 | biopolymer transport protein ExbB-related protein | | 4.8E-08 |
| VC1665 | ABC transporter, permease protein, putative |  | 2.5E-08 |
| VCA0070 | phosphate ABC transporter, periplasmic phosphate-binding protein | *pstS* | 9.7E-12 |
| VCA0267 | multidrug resistance protein D | *emrD-3* | 1.0E-09 |
| VCA0772 | tyrosine-specific transport protein | *tyrP* | 2.7E-12 |
|  |  |  |  |
| Cellular process |  |  |  |
| Locus | Function | Gene | *P* Value |
| VC0304 | guanosine-5-triphosphate,3-diphosphate pyrophosphatase | *gppA* | 1.7E-09 |
| VC1898 | methyl-accepting chemotaxis protein |  | 7.5E-13 |
| VCA0068 | methyl-accepting chemotaxis protein |  | 3.8E-09 |
| VCA1096 | chemotaxis protein CheY | *cheY-4* | 1.8E-10 |
|  |  |  |  |
| Cell Envelope |  |  |  |
| Locus | Function | Gene | *P* Value |
| VC2631 | fimbrial assembly protein PilP, putative |  | 6.2E-11 |
| VC2632 | fimbrial assembly protein PilO, putative |  | 1.9E-08 |
|  |  |  |  |
| Biosynthesis of cofactors |  |  |  |
| Locus | Function | Gene | *P* Value |
| VC0222 | lipopolysaccharide core biosynthesis protein KdtB | *kdtB* | 9.5E-08 |
|  |  |  |  |
| Central, DNA, and Energy metabolism |  |  |  |
| Locus | Function | Gene | *P* Value |
| VC1591 | oxidoreductase, short-chain dehydrogenase-reductase family |  | 1.1E-08 |
| VC0345 | DNA mismatch repair protein MutL | *mutL* | 6.4E-11 |
| VCA0155 | NADH dehydrogenase, putative |  | 3.7E-08 |
|  |  |  |  |
| Hypothetical (annotated) |  |  |  |
| Locus | Function | Gene | *P* Value |
| VC1782 | ROK family protein |  | 5.0E-08 |
| VC2750 | GGDEF family protein |  | 4.3E-09 |
| VC1322 | conserved hypothetical protein |  | 7.0E-11 |
| VCA0489 | conserved hypothetical protein | | 2.4E-08 |
| VCA0716 | conserved hypothetical protein |  | 9.1E-15 |
| VCA0948 | conserved hypothetical protein |  | 6.5E-12 |
| VCA1021 | conserved hypothetical protein |  | 1.7E-10 |
|  |  |  |  |
| Hypothetical (no annotation) |  |  |  |
| Locus | Function | Gene | *P* Value |
| VC0101 | hypothetical protein |  | 7.6E-08 |
| VC0932 | hypothetical protein |  | 3.2E-09 |
| VCA0118 | hypothetical protein |  | 6.8E-08 |
| VCA0631 | hypothetical protein |  | 8.6E-08 |
| VCA0715 | hypothetical protein |  | 5.5E-11 |
| VCA0868 | hypothetical protein |  | 2.7E-09 |
| VCA1016 | hypothetical protein |  | 2.3E-08 |
| VCA1107 | hypothetical protein |  | 2.1E-08 |
