# Supplementary material for: Transmission of Vibrio cholerae Is Antagonized by Lytic Phage and Entry into the Aquatic Environment
Source: PLoS Pathog. 2008 Oct 24;4(10):e1000187. doi: 10.1371/journal.ppat.1000187 (PMC2563029; doi:10.1371/journal.ppat.1000187)
Supplement: Table S10 — Microarray data for the patient derived V. cholerae (phage+) vs. the in vitro derived V. cholerae comparison are provided in an Excel spreadsheet. (50 KB PDF) [file ppat.1000187.s011.doc]

Supplementary Table S10. Microarray data for the patient derived *V. cholerae* (phage +) vs. the *in vitro* derived *V. cholerae* comparison are provided in an Excel spreadsheet (below). Labels for sample types (T0P*, T5P*, T24P*, T0I, T5I, T24I) are as described in Fig. 6. The median normalized intensity values and standard deviations within a given biological grouping are listed as log_2_ values. The associated *P value* (Student’s t-test) for a given comparison is listed alongside of each fold-change value. Fold changes for each pair-wise distinction calculation are listed in log_2_. Fold changes that are ≥ two-fold and have a *P value* < 0.01 are color coded red and green for induction and repression, respectively. Annotations are provided by the J. Craig Venter Institute.

For download please click on attachments at:

https://wikis.uit.tufts.edu/confluence/display/CamillLaboratory/Home

Alternatively for direct access:

https://wikis.uit.tufts.edu/confluence/pages/viewpageattachments.action?pageId=10977522
